# Insulin resistance is associated with altered amino acid metabolism and adipose tissue dysfunction in normoglycemic women

**DOI:** 10.1038/srep24540

**Published:** 2016-04-15

**Authors:** Petri Wiklund, Xiaobo Zhang, Satu Pekkala, Reija Autio, Lingjia Kong, Yifan Yang, Sirkka Keinänen-Kiukaanniemi, Markku Alen, Sulin Cheng

**Affiliations:** 1 Shanghai Jiao Tong University, Shanghai, China; 2 Department of Health Sciences, University of Jyväskylä, Jyväskylä, Finland; 3 School of Health Sciences, University of Tampere, Tampere, Finland; 4 Department of Signal Processing, Tampere University of Technology, Tampere, Finland; 5 Center for Life Course Health Research, University of Oulu, Oulu, Finland; 6 Department of Medical Rehabilitation, Oulu University Hospital, Oulu, Finland

## Abstract

Insulin resistance is associated adiposity, but the mechanisms are not fully understood. In this study, we aimed to identify early metabolic alterations associated with insulin resistance in normoglycemic women with varying degree of adiposity. One-hundred and ten young and middle-aged women were divided into low and high IR groups based on their median HOMA-IR (0.9 ± 0.4 vs. 2.8 ± 1.2). Body composition was assessed using DXA, skeletal muscle and liver fat by proton magnetic resonance spectroscopy, serum metabolites by nuclear magnetic resonance spectroscopy and adipose tissue and skeletal muscle gene expression by microarrays. High HOMA-IR subjects had higher serum branched-chain amino acid concentrations (BCAA) (p < 0.05 for both). Gene expression analysis of subcutaneous adipose tissue revealed significant down-regulation of genes related to BCAA catabolism and mitochondrial energy metabolism and up-regulation of several inflammation-related pathways in high HOMA-IR subjects (p < 0.05 for all), but no differentially expressed genes in skeletal muscle were found. In conclusion, in normoglycemic women insulin resistance was associated with increased serum BCAA concentrations, down-regulation of mitochondrial energy metabolism and increased expression of inflammation-related genes in the adipose tissue.

One of the earliest detectable defects in the metabolic continuum leading to type 2 diabetes is insulin resistance^1^. Impaired glucose homeostasis is associated with obesity^2^, but the means by which excessive adiposity induces insulin resistance and glucose intolerance remain controversial. Indeed, only about a quarter of the variance of insulin resistance is explained by BMI in the general population^3^. Studies have shown that increased risk of heart disease is independent of central obesity in individuals with metabolic syndrome^4^, and lean individuals may be as insulin resistant as those with type 2 diabetes^5^. On the other hand, not all overweight and obese individuals develop insulin resistance or other metabolic disorders^6^, suggesting that the quantitative impact of obesity *per se* on insulin sensitivity may not be as large as previously thought^7^.

Obesity-associated increase in plasma free fatty acids and accumulation of ectopic lipids are linked with the onset of peripheral and hepatic insulin resistance^8^. Dysregulated amino acid metabolism is also associated with obesity-related impaired insulin sensitivity^9^ and increased risk for future diabetes^10^. We have recently used high-through put metabolite quantification to identify metabolic differences between sedentary obese individuals with and without metabolic syndrome. We found that branched-chain and aromatic amino acids were strongly associated with insulin resistance and other metabolic risk factors, independent of fat mass and waist circumference^11^. This finding suggests that excess fat mass alone does not explain the associations of these amino acids with insulin resistance. Thus, the source of increased branched-chain and aromatic amino acids, and the mechanisms by which they might contribute to impaired insulin sensitivity remain incompletely understood.

Recent evidence suggests that obesity is associated with altered adipose tissue metabolism, which in turn affects systemic glucose homeostasis and induces insulin resistance in skeletal muscle^12^,^13^. There is little knowledge on the alterations in systemic, adipose tissue and skeletal muscle metabolism in relation to insulin resistance in normoglycemic individuals with varying degree of adiposity. Therefore, we aimed to investigate the systemic metabolite and gene expression profiles of subcutaneous adipose tissue and skeletal muscle in (fasting) normoglycemic women but differing in insulin resistance.

## Results

### Clinical characteristics of the low and high HOMA-IR groups

The high HOMA-IR group had higher body weight and BMI than the low HOMA-IR group (p < 0.01 for both, Table 1). High HOMA-IR subjects also had higher percent body fat, total and visceral fat mass (p < 0.01 for both). No significant difference in hepatic or intra-muscular fat was found. Fasting glucose concentrations were within the normoglycemic range in the majority of the subjects (92%). HOMA-IR indices were almost three times higher in individuals in the high HOMA-IR group than in those in the low HOMA-IR group. This difference was largely due to the significantly higher fasting insulin levels in the high HOMA-IR group (p < 0.001). Serum triglyceride and leptin concentrations were higher and physical activity and aerobic fitness was lower in the high HOMA-IR than low HOMA-IR group (p < 0.05 for all), but no difference in adiponectin was found. Dietary intake did not differ between the groups, nor was there a significant difference in plasma free fatty acids. No group by generation interaction was found on any of the variables.

### Serum metabolites

Serum metabolite profile analysis revealed many similarities but also significant differences between the high and low HOMA-IR groups (Table 2). There were no significant differences in the fatty acid and phospholipid (Table 2) and lipoprotein subclass concentrations (Supplementary Table S1) between the groups. However, concentrations of branched-chain amino acids (BCAA = isoleucine, leucine and valine), aromatic amino acids (AAA = phenylalanine and tyrosine), glycerol and orosomucoid were significantly higher in the high HOMA-IR group than the low HOMA-IR group (p < 0.05 for all). Only phenylalanine showed group by generation interaction. The differences between the groups remained significant after adjusting for age, total or visceral fat mass, physical activity and aerobic fitness.

To confirm that the circulating metabolite levels were not confounded by difference in adiposity, we investigated whether the differences in metabolites between the HOMA-IR groups were also consistently present in normal weight individuals. We found that serum BCAAs and orosomucoid were significantly higher in the high HOMA-IR group than the low HOMA-IR group (p < 0.05 for all) (Supplementary Table S2). No difference between the groups in the other metabolites was found. Total serum BCAA correlated with fs insulin (r = 0.388), even after adjusting for age, percent fat mass and visceral adipose tissue. The concentration of plasma free fatty acids was not associated with fs insulin (r = 0.108), HOMA-IR (r = 0.168), fat mass (r = 0.093) or visceral adipose tissue (r = 0.153) (p > 0.05 for all).

### Adipose tissue gene expression

To elucidate the metabolic pathways characterizing or contributing to insulin resistance, we studied global transcript profiles of adipose tissue and skeletal muscle. Microarray analysis revealed 1093 differentially expressed genes (688 up-regulated and 405 down-regulated) in the adipose tissue of the high HOMA-IR group (Supplementary Table S4). Kyoto Encyclopedia of Genes and Genomes (KEGG) enrichment analysis of the differentially expressed genes (p < 0.05) identified 9 down-regulated and 15 up-regulated pathways.

The up-regulated pathways in the adipose tissue revealed increased activation of immune response involving both innate and adaptive immune systems (Table 3). Lysosome was the most up-regulated pathway (p = 5.6 × 10^−13^), with contributions by genes involving all aspects of lysosome biogenesis and function, including structural genes (*LAMP1, LAMP2* and *LAPTM5*), several lysosomal acid hydrolases and transport proteins of lipids and cholesterol (*MCOLN1* and *NPC2*) as well as proteins required for lysosome acidification (*ATP6V0D2* and *ATP6AP1*). Several chemokines (e.g., *CCL2, CCL3, CCL4* and *CCL5*), which are produced by innate immune cells, and also by pre-adipocytes and mature adipocytes^14^ were also present in the up-regulated pathways. Consistently, leukocyte trans-endothelial migration, Fc gamma R-mediated phagocytosis, toll-like receptor signaling, and the complement system, (complement *C1* [*C1S, C1QA, C1QB, C1QC*]) were up-regulated in the high HOMA-IR subjects. Up-regulation of the B cell receptor signaling pathway was also observed, providing further evidence for the involvement of the adaptive immune system and infiltration of inflammatory cells to the adipose tissue in insulin resistance.

The most down-regulated pathway in the adipose tissue was valine, leucine and isoleucine degradation (p = 1.1 × 10^−7^, Table 4). The genes that mapped to this pathway encode the cytosolic and mitochondrial components of the pathway and included both genes common to the degradation of all BCAAs, namely isoleucine, leucine and valine (*BCAT1, BCKDHB*) and those specific for the degradation of leucine (*MCC2, AUH*), isoleucine (*PCCA, PCCB*) and valine (*HIBADH, ALDH6A1*). Further, we found that systemic insulin resistance was also associated with significant down-regulation of the genes encoding proteins that play important roles in cellular energy homeostasis. These included a master regulator of mitochondrial biogenesis and function, peroxisome proliferator-activated receptor-gamma coactivator-1α (*PPARGC1A*) and mitochondrial acetyl-coenzyme A carboxylase beta (*ACACB*), which is an important regulator of fatty acid oxidation and synthesis. Further, peroxisome proliferator-activated receptor alpha (*PPARA*), which promotes the uptake, utilization, and catabolism of fatty acids, was also down-regulated in the high HOMA-IR group. Accordingly, down-regulation of the fatty acid degradation pathway and tricarboxylic acid cycle (TCA cycle) was observed. Other down-regulated pathways involved aromatic amino acid (phenylalanine and tryptophan) and short-chain fatty acid (propanoate) metabolism, and lysine biosynthesis.

**Figure 1:**
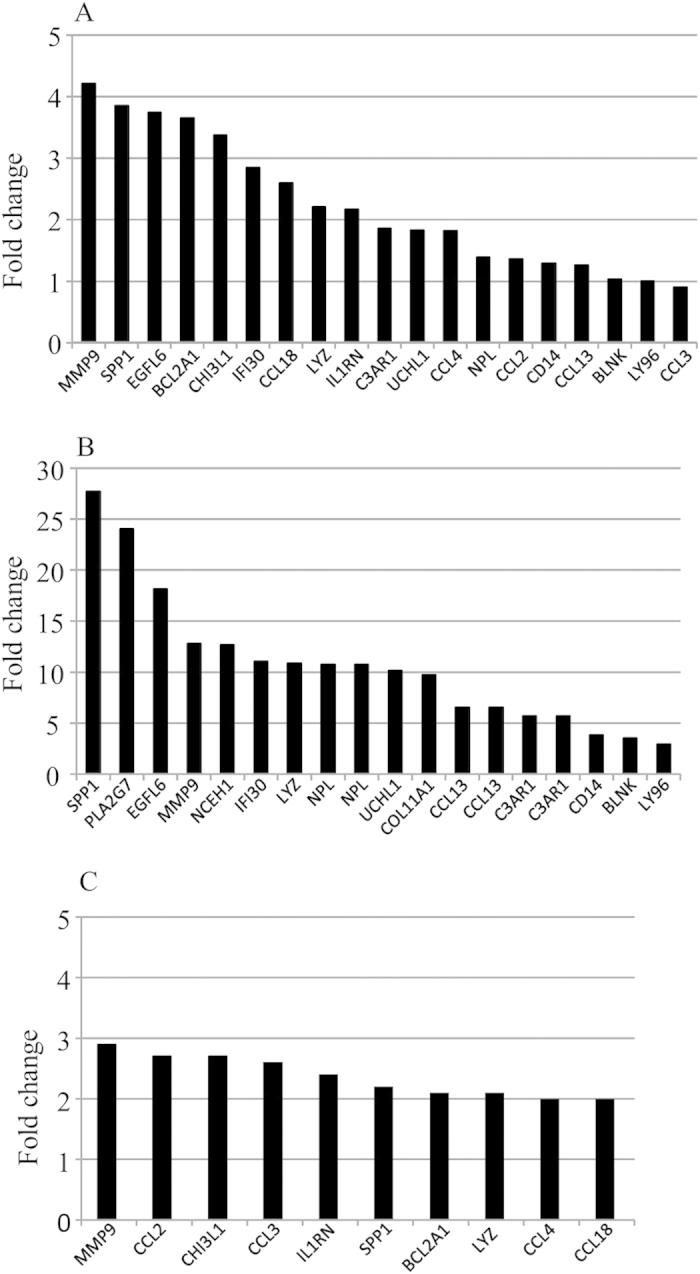
Up-regulated inflammation-related genes in the adipose tissue in insulin resistant compared to insulin sensitive group. The y-axis shows the fold change of gene expression of up-regulated genes in high HOMA-IR subjects of the present study (**A**) and obese insulin resistant subjects in the study of Soronen *et al*. (**B**)^15^ and Hardy *et al*.^16^ (**C**).

To ensure our observations were not biased, we validated our results with two other independent experiments in obese insulin-resistant subjects by using gene expression omnibus (GEO) and the GSE26637^15^ and GSE20950^16^ and data sets, respectively. The up-regulated inflammation-related genes in the adipose tissue were similar in our study than in the study of Soronen *et al*.^15^ and Hardy *et al*.^16^ (Fig. 1). In addition, the inflammation and energy metabolism-related pathways in the adipose tissue were similarly up- and down-regulated than in obese insulin resistant women in the study of Soronen *et al*.^15^.

**Figure 2:**
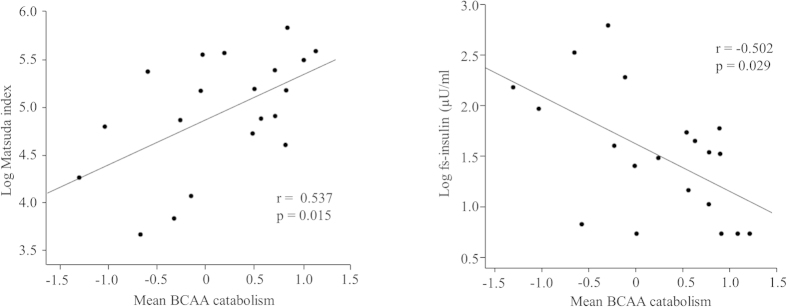
Correlations between the mean centroids of the BCAA catabolism pathway and fs-insulin and insulin sensitivity (Matsuda index). The values of fs-insulin and the Matsuda index were transformed to normal distribution by natural logarithms. Each dot represents an individual and the line is a linear regression fit line.

**Figure 3:**
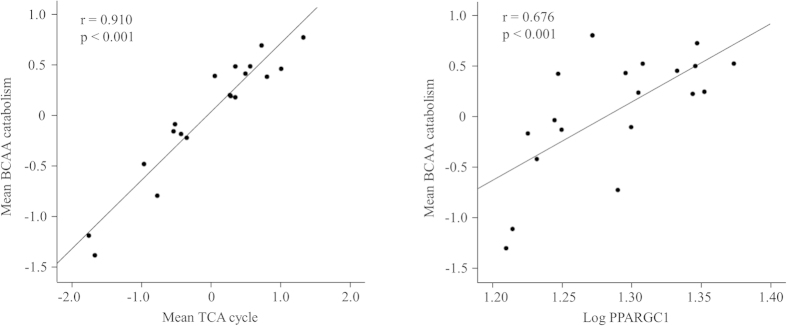
Correlation between the mean centroids of the BCAA catabolism pathway and the TCA cycle and PPARGC1A. Each dot represents an individual and the line is a linear regression fit line.

We then assessed the associations between adipose tissue gene expressions and clinical traits in non-obese individuals. Characteristics of the study participants are presented in Supplementary Table S3. The mean centroid of the BCAA catabolism pathway in the adipose tissue was associated with insulin sensitivity (Matsuda index) and fs-insulin (p < 0.05 for all) (Fig. 2). These associations remained significant after adjusting for age and percent fat mass or visceral fat mass. The BCAA catabolism pathway correlated closely with mitochondrial respiration and biogenesis, i.e., with the TCA cycle and PPARGC1A (p < 0.001) (Fig. 3). Maximum oxygen uptake (VO_2max_) correlated with the BCAA catabolism (r = 0.543) and the TCA cycle (r = 0.522) (p < 0.05 for all). Inflammation pathways were coordinately up-regulated with insulin resistance and adipokines, e.g., the chemokine signaling pathway correlated with insulin sensitivity (Matsuda index) (r = −0.807), fs-insulin (r = 0.858), fs-adiponectin (r = −0.598) and leptin (r = 0.428) (p < 0.001 for all). The chemokine signaling pathway displayed significant associations with the TCA cycle and BCAA catabolism (r = −0.812 and r = −0.788, respectively, p < 0.001 for both).

### Skeletal muscle gene expression and signaling protein phosphorylation

Unexpectedly, when the transcriptomic data of skeletal muscle were studied, no differentially expressed genes were found between the low and high HOMA-IR samples. However, since skeletal muscle is the primary site of insulin-stimulated glucose disposal, we studied whether there were differences in the phosphorylation levels of several proteins related to glucose uptake, insulin signaling and mitochondrial energy metabolism. No differences in the phosphorylation levels of insulin receptor β or its downstream target Akt were found (Supplementary Figure S1). The level of phosphorylated AS160, which promotes translocation of glucose transporters to the cell membrane, was also similar between the groups. In addition, no differences in the expression of mitochondrial respiratory chain complex subunits, namely ATP5A, UQCRC2, MTCO1, SDHB and NDUFF88 between the groups were found (Supplementary Figure S1).

## Discussion

In this study with young and middle-aged normoglycemic women, we found that insulin resistance was associated with increased serum BCAA levels, independent of obesity. Consistent with this, we found a significant down-regulation of genes related to BCAA catabolism and mitochondrial energy metabolism, concurrently with increased expression of inflammation-related genes in the adipose tissue.

Plasma free fatty acids are commonly elevated in obese individuals due to increased adipose tissue lipolysis^17^. Elevated circulating free fatty acids may accumulate in other insulin-responsive tissues, such as skeletal muscle and liver (where they interfere with insulin signaling and cause insulin resistance)^18^. However, we found that in normoglycemic women with varying degree of adiposity, insulin resistance was not associated with increased plasma free fatty acids, but instead with increased serum amino acid concentrations. Our observation partly agrees with a recent study in normal weight subjects discordant for insulin sensitivity^19^, and a meta-analysis by Karpe *et al*.^20^. It is tempting, therefore, to suggest that the contribution of circulating free fatty acids to insulin resistance may be relatively small.

Increased serum BCAA concentrations have been associated with obesity-related insulin resistance in earlier studies^21^,^22^,^23^. Our results suggest that perturbations in systemic BCAA homeostasis are related to insulin resistance rather than to obesity *per se,* since significant difference in these amino acids was observed between the high and low HOMA-IR groups also in normal weight individuals (Table S2). The average difference in total BCAA between low and high HOMA-IR groups was ~14% in whole study population and ~10% in normal weight individuals. Whether such difference is physiologically meaningful is not clear. However, Sunny *et al*.^24^ recently demonstrated that insulin-stimulated increases (10–20%) in plasma BCAA correlated significantly with insulin resistance indices in humans. They concluded that such small but chronic increase in circulating BCAA with insulin resistance may be sufficient to disrupt signaling events in the mitochondria of the muscle and liver thereby contributing to mitochondrial dysfunction. However, our study cannot show temporal relationships, although our results are compatible with previous studies^25^,^26^, which have suggested that BCAAs associate with insulin resistance.

It is unclear why serum BCAA is elevated in obesity and insulin resistance. Differences in diet^23^, protein turnover (muscle loss)^27^ or liver fat (fatty liver disease) can affect circulating levels of amino acids. However, in our study no difference in diet (protein intake), fat-free mass or liver fat content was observed between the low and high HOMA-IR groups. The positive correlation of BCAA with insulin concentration in our study is line with earlier reports, which have suggested that these amino acids may stimulate insulin secretion from the pancreas^21^,^28^,^29^. On the other hand, elevated insulin may increase circulating BCAA, possibly by attenuating BCAA catabolism in different tissues as suggested by Sunny *et al*.^24^. Indeed, obesity-related increases in circulating BCAAs have been associated with decreased BCAA catabolism in adipose tissue^30^. Here, we showed significant down-regulation of the BCAA degradation pathway genes in the adipose tissue of normoglycemic subjects with high insulin resistance. The fact that there was no difference in average BMI or percent body fat between the low and high HOMA-IR groups suggests that down-regulation of the BCAA catabolism pathway was not attributable to adiposity alone. Thus, the decrease in the BCAA catabolism can probably be ascribed to reduced mitochondrial respiration and biogenesis (as indicated by the close correlation of the BCAA catabolism with the TCA cycle and PPARGC1A genes). Since physical activity and aerobic fitness are known to improve insulin sensitivity and energy metabolism^31^, it is possible that differences in physical activity and aerobic fitness may have amplified the observed differences in gene expression between the low and high HOMA-IR groups. The close correlation between VO_2max_ and BCAA catabolism and the TCA cycle further support this notion.

Growing evidence indicates that obesity-associated low-grade inflammation of adipose tissue contributes to the development of insulin resistance^32^. Our results complement this notion by showing that up-regulated inflammation-related genes were closely associated with insulin resistance, serum adiponectin and leptin also in normoglycemic individuals, even after adjusting for measures of adiposity. Earlier studies have demonstrated that plasma adiponectin and leptin levels are associated with insulin resistance independent of fat mass^33^,^34^,^35^, and they may play a key role in the regulation of inflammation and immunity^36^. In line with earlier studies^37^,^38^, we found that the innate inflammatory component related to macrophages was coordinately up-regulated with insulin resistance. The physiological role of macrophages is probably to clear adipose debris through the process of phagocytosis and activate the adaptive immune system^39^. Accordingly, we found that in terms of over-expressed genes the most up-regulated pathways in the adipose tissue were lysosome and phagosome pathways. These findings, (together with activation and infiltration of lymphocytes, a toll-like receptor signaling pathway, and the complement system) suggest a state of chronic low-grade inflammation in the adipose tissue. Furthermore, the chronic inflammation may also in part explain the observed impairments in adipose tissue energy metabolism (as indicated by the close inverse correlation of chemokine signaling genes with BCAA catabolism and the TCA cycle genes).

Impaired insulin-mediated skeletal muscle glucose uptake^40^ and intramyocellular lipid concentrations^41^ are major contributors to insulin resistance and type 2 diabetes. In our study, intramuscular triglycerides were not increased in subjects with high HOMA-IR. Consistent with this, no aberrant gene expression in individuals with high HOMA-IR was found. To confirm these findings, we further studied whether there were differences in the phosphorylation levels of several signaling proteins related to glucose metabolism. No significant differences in the phosphorylation levels of insulin receptor β and its downstream target Akt, were found, nor was there any significant difference in the level of phosphorylated AS160, which promotes translocation of glucose transporters to the cell membrane. Previous studies have reported reduced muscle transcript levels related to oxidative metabolism in diabetic individuals compared to healthy controls^42^. We found no difference in mitochondrial respiratory chain complex subunits between the low and high HOMA-IR groups. These findings suggest that in the fasting state glucose and mitochondrial energy metabolism is not significantly altered in the skeletal muscle in early stages of insulin resistance. However, since both acute hyperinsulinemia^43^ and hyperglycemia^44^ have been shown to induce transcriptional and translational regulation of glucose and energy metabolism in the skeletal muscle, it may be that significant differences could exist during hyperinsulinemic-euglycemic clamp, glucose challenge or mixed meal feeding.

This study has some limitations. First, the number of participants in our study was relatively small and consisted solely of women. Despite this we were able to identify statistically significant differences in serum metabolites and gene expressions in adipose tissue between the groups. In addition, the participants were carefully selected in order to minimize confounding factors and genetic variability. Furthermore, gene expression in the adipose tissue of participants with high HOMA-IR was validated by two independent experiments with non-obese type 2 diabetic and obese insulin-resistant subjects. Thus, we believe that our results are not biased and that the gene expression data can be viewed with confidence. Finally, the majority of the participants were within the normal fasting glycemic range and exclusion of those with impaired fasting glucose did not change the results. This gave us the possibility to identify the biomarkers associated with systemic insulin resistance in its early stage.

Our data demonstrate that serum fatty acids, intra-myocellular lipids and liver fat content were not elevated in normoglycemic women with high HOMA-IR. Instead, we show that impaired insulin sensitivity was associated with a significant increase in serum BCAA concentration, up-regulation of inflammation-related genes and down-regulation of genes related to BCAA catabolism and mitochondrial energy metabolism in adipose tissue. These findings suggest that adipose tissue inflammation and mitochondrial dysfunction may be early events in the development of systemic insulin resistance. Further studies are needed to determine the initial factor(s) that trigger the transcriptional changes that lead to these metabolic alterations.

## Materials and Methods

### Study subjects

This article is part of a large family study with 282 participating families and has been described elsewhere^45^. A subgroup of families (n = 74), comprising 222 individuals (daughter, mother and father) with no type I/II diabetes or family history (first degree relative) of diabetes, cardiac diseases, autoimmune diseases or major liver (cancer, hepatitis) diseases were contacted by letter for an additional study aimed at identifying biomarkers associated with insulin resistance and liver fat accumulation. A total of 184 individuals responded to our invitation, of whom 163 (53 fathers, 53 mothers and 57 daughters) attended the laboratory tests. For this report, all the fathers were excluded in order to reduce the variability in genetic architecture, leaving only the mothers and daughters (mothers = 53 and daughters = 57). Mothers and daughters did not differ in measures of adiposity, insulin resistance, serum triglycerides, fatty acids or amino acids. Therefore, all data including the metabolome and microarray data were pooled before the phenotypic analysis and all results were adjusted for age and familiarity. Further, to minimize the metabolic alterations occurring at different stages of the menstrual cycle, blood samples were collected from the women with regular menses between 2 and 5 days after (menstruation). Twenty-two participants were in early post menopause but none were on hormonal replacement therapy. Including or excluding these participants did not influence the results. All subjects were clinically euthyreotic. The study protocol was approved by the ethics committee of the Central Finland Health Care District. A written informed consent was obtained from all participants, and all experiments were performed in accordance with relevant guidelines and regulations.

### Methods. 

A detailed description of the background information and methods are provided in supplementary text S1. In short, background information including medical history, current health status and physical activity was collected via self-administered questionnaires. Food consumption and intakes of total energy and energy-yielding nutrients were assessed from three-day food records. All measurements were performed in the morning after overnight fasting. Venous blood samples were obtained for the analyses of glucose, insulin, non-esterified fatty acids, leptin, and adiponectin. The HOMA-IR index (homeostatic model assessment of insulin resistance) was calculated as (fasting glucose x fasting insulin/22.5). According their HOMA-IR values (median = 1.57), the subjects were divided into low (n = 55) and high (n = 55) groups. Body composition was assessed by DXA, subcutaneous and intra-abdominal adipose tissue by MRI^46^, and ectopic fat of liver, muscle intra-myocellular lipid (IMCL) and extra-myocellular lipid (EMCL) by ^1^HMRS^47^. Serum metabolites were assessed by NMR spectroscopy^48^. Maximum oxygen uptake (VO_2max_, ml/kg/min) was assessed by a bicycle ergometer test. In addition, superficial abdominal subcutaneous adipose tissue and skeletal muscle (vastus lateralis) biopsies were obtained from 24 individuals to assess the differences in global gene expression profiles and muscle protein expression between the low and high HOMA-IR groups. Furthermore, a 75-g oral glucose tolerance test (OGTT) was performed for subjects with tissue biopsies to assess whole body insulin sensitivity^49^. Microarray measurements were analyzed by using the Robust Multiarray Averaging (RMA) algorithm in the Bioconductor R package affy^50^,^51^,^52^. The Limma R package was used for differentially expressed genes (DEGs). Raw p values were adjusted to control for the false discovery rate (FDR) using the method of Benjamini and Hochberg^53^ (for more detailed information on microarray and gene enrichment analysis see supplementary text S1).

### Statistical methods

Before each analysis, continuous data were checked for normality by Shapiro-Wilk’s test using PASW statistics version 21 (IBM Corporation, USA). If data were not normally distributed, their natural logarithms were used. Clinical characteristics and serum metabolites were compared using an independent-samples t-test. Since the data were from a family study, the familiarity (genetic and environmental (household) similarity) was controlled by using linear mixed model to compare levels of the outcome variables between the low and high HOMA-IR groups. Contrast tests were used in mixed models to assess the effect of generation while controlling for dependency among family members with random effects. P-values were adjusted to control for the false discovery rate (FDR) using the method of Benjamini and Hochberg when comparing metabolites between the low and high HOMA-IR groups^53^. Pearson correlation analysis was used to determine the relationship between clinical characteristics, serum metabolites and adipose tissue gene expression. Statistical significance was set at p < 0.05.

## Additional Information

**How to cite this article**: Wiklund, P. *et al*. Insulin resistance is associated with altered amino acid metabolism and adipose tissue dysfunction in normoglycemic women. *Sci. Rep.* **6**, 24540; doi: 10.1038/srep24540 (2016).

## Supplementary Material

Supplementary Information

## Figures and Tables

**Table 1:** General characteristics in the low and high HOMA-IR groups (MIXED model estimated marginal means with 95% confidence intervals are given taking into account genetic similarity and shared environment (daughter and mother) and contrast estimates’ p-values were used to localize the significant differences between the two groups and group by generation interaction).

	low HOMA-IR (n = 55)		high HOMA-IR (n = 55)		p-value	Group by generation
	Mean	95% CI	Mean	95% CI		
Anthropometry						
Age (years)	35.1	(35.9, 36.3)	36.0	(35.0, 36.9)	0.269	0.096
Height (cm)	165.7	(163.9, 167.7)	166.1	(164.5, 167.6 )	0.837	0.562
Weight (kg)	61.3	(58.4, 64.1)	67.3	(65.1, 69.5)	0.001	0.604
BMI (weight(kg)/height(m)2)	22.3	(21.4, 23.2)	24.4	(23.7, 25.1)	0.002	0.497
Body composition						
Percent body fat	29.5	(27.3, 31.6)	35.0	(32.9, 37.1)	0.001	0.209
FFM (kg)	40.6	(39.3, 42.0)	40.7	(39.6, 41.8)	0.944	0.417
FM (kg)	17.8	(15.5, 20.1)	23.7	(21.9, 25.5)	0.001	0.266
VAT (kg)	0.5	(0.47, 0.62)	0.7	(0.59, 0.70)	0.044	0.164
Liver fat (%)	2.5	(0.9, 4.1 )	2.7	(1.5, 4.0)	0.787	0.577
IMCL (%)	0.2	(0.16, 0.25)	0.2	(0.17, 0.23)	0.796	0.261
EMCL (%)	0.2	(0.15, 0.31)	0.3	(0.27, 0.39)	0.068	0.925
Metabolic biomarkers						
fs-glucose (mmol/l)	5.2	(5.0, 5.4)	5.5	(5.3, 5.6)	0.042	0.982
fs-insulin (μU/ml)	4.1	(3.0, 5.1)	9.9	(9.0, 10.8)	<0.001	0.110
HOMA-IR	0.9	(0.6, 1.2)	2.4	(2.2, 2.7)	<0.001	0.112
Lipids						
FFA (mmol/l)	3.6	(3.0, 4.2)	4.0	(3.5, 4.5)	0.308	0.968
Triglycerides (mmol/l)	0.9	(0.7, 1.0)	1.1	(1.0, 1.2)	0.013	0.250
Adipokines						
Leptin (ng/ml)	14.6	(9.0, 20.2)	27.4	(23.1, 31.7)	0.001	0.490
Adiponectin (μg/ml)	10.4	(18.2, 20.6)	10.1	(8.4, 11.8)	0.830	0.375
Diet						
Energy (kcal)	1840.0	(1690, 1990)	1780.0	(1650, 1900)	0.523	0.583
Protein (E%)	17.7	(16.3, 18.8)	18.3	(17.3, 19.3)	0.324	0.545
Fat (E%)	34.5	(31.4, 36.0)	32.3	(30.1, 34.0)	0.283	0.564
Carbohydrates (E%)	47.8	(44.8, 49.5)	49.4	(46.4, 50.3)	0.414	0.868
Physical activity and fitness						
LTPA (hours/week)	4.5	(3.8, 5.2)	3.6	(3.0, 4.1)	0.049	0.466
VO_2max_ (ml/kg/min)	41.1	(36.8, 45.5)	35.1	(31.3, 38.9)	0.042	0.540

FFM = fat-free mass; FM = fat mass; SAT = subcutaneous adipose tissue; VAT = visceral adipose tissue; IMCL = intra-myocellular lipids; EMCL = extra-myocellular lipids; HOMA-IR = homeostatic model assessment of insulin resistance; OGTT = oral glucose tolerance test; Matsuda index = insulin sensitivity index; FFA = free fatty acids; E% = percentage of total energy intake; LTPA = leisure-time physical activity; VO_2max_ = maximum oxygen uptake.

**Table 2:** Serum low-molecular weight metabolites and lipid extract constituents in the low and high HOMA-IR groups(MIXED model estimated marginal means with 95% confidence intervals are given taking into account genetic similarity and shared environment (daughter and mother) and contrast estimates’ p-values were used to localize the significant differences between the two groups and group by generation interaction).

	low HOMA-IR (n = 55)		high HOMA-IR (n = 55)		pBH	Group by generation
	Mean	95% CI	Mean	95% CI		
Low-molecular weight metabolites						
betahydroxybutyrate	0.049	(0.033, 0.066	0.061	(0.046, 0.077)	0.357	0.253
acetate	0.039	(0.036, 0.042)	0.043	(0.040, 0.046)	0.081	0.785
acetoacetate	0.038	(0.032, 0.044)	0.034	(0.029, 0.040)	0.961	0.793
alanine	0.374	(0.353, 0.394)	0.399	(0.380, 0.418)	0.261	0.305
citrate	0.098	(0.092, 0.104)	0.102	(0.096, 0.108)	0.406	0.923
creatinine	0.049	(0.046, 0.053)	0.051	(0.048, 0.055)	0.197	0.311
glutamine	0.511	(0.494, 0.529)	0.533	(0.517, 0.550)	0.685	0.174
glycerol	0.056	(0.047, 0.064)	0.074	(0.066, 0.082)	0.003	0.134
glycine	0.257	(0.239, 0.276)	0.276	(0.259, 0.292)	0.685	0.211
orosomucoid	1.225	(1.168, 1.281)	1.344	(1.291, 1.397)	0.045	0.443
histidine	0.050	(0.047, 0.053)	0.056	(0.053, 0.058)	0.362	0.329
isoleucine	0.038	(0.035, 0.041)	0.043	(0.040, 0.046)	0.040	0.959
leucine	0.061	(0.057, 0.064)	0.069	(0.066, 0.073)	0.005	0.813
valine	0.154	(0.144, 0.163)	0.174	(0.165, 0.183)	0.014	0.351
BCAAsum	0.253	(0.237, 0.268)	0.288	(0.273, 0.302)	0.009	0.666
phenylalanine	0.061	(0.058, 0.064)	0.066	(0.063, 0.069)	0.035	0.045
tyrosine	0.041	(0.037, 0.044)	0.047	(0.044, 0.050)	0.030	0.089
pyruvate	0.068	(0.061, 0.075)	0.077	(0.070, 0.083)	0.064	0.915
lactate	0.891	(0.799, 0.984)	0.967	(0.877, 1.057)	0.253	0.101
urea	0.052	(0.045, 0.058)	0.049	(0.043, 0.055)	0.731	0.131
Lipid extract constituents						
esterified cholesterol	2.691	(2.528, 2.855)	2.831	(2.675, 2.987)	0.147	0.422
free cholesterol	1.020	(0.952, 0.1087)	1.097	(1.032, 1.161)	0.127	0.386
omega3 fatty acids	0.361	(0.316, 0.406)	0.369	(0.326, 0.411)	0.699	0.551
omega6 fatty acids	2.789	(2.626, 2.952)	2.920	(2.824, 3.136)	0.328	0.683
omega7and 9 fatty acids	4.918	(4.510, 5.325)	5.422	(5.032, 5.812)	0.253	0.446
total fatty acids	8.068	(7.505, 8.630)	8.770	(8.233, 9.308)	0.259	0.699
linoleic acid	2.303	(2.157, 2.449)	2.501	(2.362, 2.641)	0.328	0.738
polyunsaturated fatty acids	1.647	(1.493, 1.801)	1.690	(1.543, 1.837)	0.792	0.747
docosahexanoic acid	0.143	(0.122, 0.164)	0.145	(0.125, 0.165)	0.716	0.872
monounsaturated fatty acids	2.183	(1.976, 2.389)	2.429	(2.232, 2.626)	0.253	0.370
total phosphoglycerides	0.620	(0.567, 0.673)	0.671	(0.620, 0.721)	0.685	0.763
phosphocholines	1.526	(1.413, 1.639)	1.622	(1.514, 1730)	0.685	0.700
sphingomyelines	0.202	(0.187, 0.216)	0.207	(0.193, 0.220)	0.685	0.520
omega3/total fatty acid ratio	4.453	(4.066, 4.840)	4.165	(3.795, 4.536)	0.458	0.173
omega6/total fatty acid ratio	34.87	(33.80, 35.95)	34.40	(33.36, 35.42)	0.676	0.206
omega7and9/total fatty acid ratio	60.67	(59.61, 61.73)	61.45	(60.43, 62.46)	0.458	0.076
fatty acid length	17.92	(17.84, 18.00)	17.94	(17.87, 18.01)	0.857	0.264

P-values are adjusted for multiple comparisons using Benjamin-Hochberg correction. All metabolites are in mmol/l.

**Table 3:** Up-regulated pathways in the adipose tissue of high HOMA-IR group.

P-value	Count	Size	Pathway name	Gene Names
5.6 × 10^−13^	36	121	Lysosome	*ACP5, AP1B1, ARSB, ATP6AP1, ATP6V0B, ATP6V0D2, CD68, CTSA, CTSB, CTSG, CTSH, CTSS, CTSZ, DNASE2B, FUCA1, GAA, GBA, GLA, GLB1, GM2A, GUSB, HEXB, LAMP1, LAPTM5, LGMN, MAN2B1, MCOLN1, NAGA, NPC2, PLA2G15, PPT1, PSAP, LAMP2, SLC11A2, SMPD1, TCIRG1*
2.2 × 10^−5^	28	156	Phagosome	*ACTB, ACTG1, ATP6AP1, ATP6V0B, ATP6V0D2, ATP6V1B2, C1R, CD14, CLEC7A, CORO1A, CTSS, CYBA, FCGR2A, FCGR2B, FCGR3A, ITGB2, ITGB5, LAMP1, MARCO, MSR1, NCF2, NCF4, SEC61A1, TCIRG1, TUBA1C, TUBB2A, TUBB2B, VAMP3*
2.0 × 10^−4^	32	189	Chemokine signaling pathway	*ADCY6, ADCY7, ADRBK2, ARRB2, CCL13, CCL18, CCL19, CCL2, CCL22, CCL3, CCL4, CCL5, CCR1, CXCL10, CXCL16, CXCR4, DOCK2, FGR, GNAI1, GNB4, GNG2, GRB2, HCK, PIK3R5, PREX1, PRKCB, PRKX, RAC2, STAT2, STAT3, TIAM1, VAV1*
7.4 × 10^−4^	21	117	Leukocyte transendothelial migration	*ACTB, ACTG1, ACTN1, CXCR4, CYBA, EZR, F11R, GNAI1, ICAM1, ITGAL, ITGB2, MMP9, MSN, MYL9, NCF2, NCF4, PIK3R5, PRKCB, RAC2, THY1, VAV1*
2.5 × 10^−3^	16	95	Fc gamma R-mediated phagocytosis	*ARF6, CFL2, DOCK2, FCGR2A, FCGR2B, FCGR3A, HCK, LAT, PIK3R5, PPAP2B, PRKCB, PTPRC, RAC2, SCIN, SYK, VAV1*
1.9 × 10^−3^	17	102	Toll-like receptor signaling pathway	*CCL3, CCL4, CCL5, CD14, CD86, CXCL10, IRAK1, LBP, LY96, MAP2K3, MAPK10, PIK3R5, SPP1, TLR1, TLR5, TLR7, TLR8*
2.8 × 10^−3^	10	65	Glycolysis/Gluconeogenesis	*ADH1B, ALDH2, ALDH3B1, ALDH7A1, ENO1, GALM, HK3, PDHB, PFKP, PGAM2*
7.8.0 × 10^−3^	5	17	Renin-angiotensin system	*AGTR1, ANPEP, CTSA, CTSG, NLN*
3.3 × 10^−3^	6	17	Other glycan degradation	*FUCA1, FUCA2, GBA, GLB1, HEXB, MAN2B1*
1.3 × 10^−2^	12	75	B cell receptor signaling pathway	*BLNK, DAPP1, FCGR2B, GRB2, NFKBIE, PIK3AP1, PIK3R5, PRKCB, PTPN6, RAC2, SYK, VAV1*
1.3 × 10^−2^	5	19	Glycosaminoglycan degradation	*ARSB, GLB1, GUSB, HEXB, HPSE*
1.1 × 10^−2^	9	48	Amino sugar and nucleotide sugar metabolism	*CHI3L1, CHIT1, GNE, GNPDA1, HEXB, HK3, NAGK, NPL, PMM1*
1.7 × 10^−2^	11	69	Complement and coagulation cascades	*C1QA, C1QB, C1QC, C1R, C1S, C3AR1, C5AR1, F13A1, PLAU, PLAUR, SERPINE1*
3.5 × 10^−2^	17	136	Natural killer cell mediated cytotoxicity	*BID, CD48, FCER1G, FCGR3A, GRB2, HCST, ICAM1, ITGAL, ITGB2, LAT, PIK3R5, PRKCB, PTPN6, RAC2, SYK, TYROBP, VAV1*
1.2 × 10^−2^	25	200	Focal adhesion	*ACTB, ACTG1, ACTN1, BIRC3, CCND2, COL6A1, COL6A2, COL6A6, FLNA, GRB2, ITGB5, LAMB3, MAPK10, MYL9, PDGFA, PIK3R5, PPP1CA, PRKCB, PTEN, RAC2, SPP1, TNC, VAV1, VEGFA, ZYX*

Count = Amount of differentially expressed genes that map in pathway. Size = Total amount of genes involved in pathway.

**Table 4:** Down-regulated pathways in the adipose tissue of high HOMA-IR group.

P-value	Count	Size	Pathway name	Genes
1.1 × 10^−7^	17	44	Valine, leucine and isoleucine catabolism	*ACADM, ACADSB, ALDH2, ALDH6A1, ALDH7A1, AUH, BCAT1, BCKDHB, DLD, HADH, HIBADH, IL4I1, MCCC2, MUT, OXCT1, PCCA, PCCB*
3.7 × 10^−4^	10	32	Proprionate metabolism	*ACACB, ACADM, ACSS3, ALDH2, ALDH6A1, ALDH7A1, MUT, PCCA, PCCB, SUCLG2*
2.9 × 10^−3^	6	17	Phenylalanine metabolism	*ALDH3B1, AOC2, IL4I1, MAOB, MIF, PRDX6*
4.4 × 10^−3^	10	43	Fatty acid degradation	*ACADL, ACADM, ACADSB, ADH1B, ADH1C, ALDH2, ALDH7A1, CPT1A, HADH, PECI*
1.2 × 10^−2^	9	42	Tryptophan metabolism	*ALDH2, ALDH7A1, CAT, CYP1B1, HADH, IL4I1, KMO, KYNU, MAOB*
1.6 × 10^−2^	7	30	Citrate cycle (TCA cycle)	*DLD, DLST, IDH3B, PC, PDHB, SDHB, SUCLG2*
2.9 × 10^−2^	8	41	Tyrosine metabolism	*ADH1B, ADH1C, ALDH3B1, AOC2, COMT, IL4I1, MAOB, MIF*
1.7 × 10^−2^	2	3	Lysine biosynthesis	*AASS, ALDH7A1*
4.7 × 10^−2^	4	18	Glyoxylate and dicarboxylate metabolism	*HYI, MUT, PCCA, PCCB*
1.9 × 10^−2^	12	68	Adipocytokine signaling pathway	*ACACB, ADIPOQ, ADIPOR1, CPT1A, MAPK10, NFKBIE, PPARA, PPARGC1A, PRKAG1, SLC2A4, STAT3, TNFRSF1B*

Count = Amount of differentially expressed genes that map in pathway. Size = Total amount of genes involved in pathway.
