# Supplementary material for: Insulin resistance is associated with altered amino acid metabolism and adipose tissue dysfunction in normoglycemic women
Source: Sci Rep. 2016 Apr 15;6:24540. doi: 10.1038/srep24540 (PMC4832240; doi:10.1038/srep24540)

Insulin resistance is associated with altered amino acid metabolism and adipose tissue dysfunction in normoglycemic women.

Petri Wiklund, Xiaobo Zhang, Satu Pekkala, Reija Autio, Lingjia Kong, Yifan Yang, Sirkka Keinänen-Kiukaanniemi, Markku Alen, and Sulin Cheng

Background information

Background information including medical history and current health status was collected via self-administered questionnaires. Food consumption and intakes of total energy and energy yielding nutrients were assessed from three day food records and analyzed using Micro-Nutrica software developed by the Social Insurance Institution of Finland and updated with data for new foodstuffs by the study nutritionist. Leisure time physical activity (LTPA) of hours/week (participating in exercise such as walking, jogging, running, gym fitness, ball games, swimming, etc.) and physical inactivity hours per day (including lying down and sitting time) were evaluated using a validated self-administrated physical activity questionnaire described previously [1].

Anthropometric, ectopic fat content and abdominal fat mass assessment

All measurements were performed after an overnight fasting. Subjects were weighed with light clothes and without shoes. Weight was determined within 0.1 kg for each subject using an electronic scale and was calibrated before each measurement session. Height was determined using a fixed wall-scale measuring device to the nearest 0.1 cm. Body mass index (BMI) was calculated as weight (kilograms) per height (meters)^2. Whole body fat mass (FM) and fat free mass (FFM) was assessed by a Dual-energy X-ray absorptiometry (DXA

Prodigy, GE Lunar Corp., Madison, WI USA). Two repeated measurements of FM and FFM showed a coefficient of variation (CV) of 2.2% and 1.0% respectively in this study [2].

Abdominal region and liver were scanned using a 1.5 Tesla MR-scanner (GE Sigma CV/i, General Electric Healthcare, Waukesha, WI, USA). Abdominal visceral adipose tissue (VAT) was quantified from a single slice image at the level of the L2-L3 intervertebral disc using the OsiriX software (OsiriX Foundation, Geneva, Switzerland). The results were converted into tissue fat mass in kilograms taking into account slice thickness and an adipose tissue density of 0.9196 g/ml [3]. Liver fat content was assessed by 1HMRS with a PRESS sequence and was analyzed using the Linear Combination of Model spectra software which is generally considered to be the gold standard for in-vivo spectroscopy analysis [4].

Muscle intra-myocellular lipid (IMCL) and extra-myocellular lipid (EMCL) from the tibialis anterior muscle were measured using a similar 1H MRS method with a surface coil placed over the middle part of the muscle [5]. In order to obtain maximal IMCL and EMCL separation the tibialis anterior muscle was aligned as good as possible with the direction of the magnetic field and the voxel was placed parallel to the muscle fibers [5].

Fitness test

Maximum oxygen uptake (VO2max, ml/kg/min) was assessed by bicycle ergometer. During tests, heart rates were assessed using ECG and respiratory gases and ventilation was measured using respiratory gas analyzer VIASYS (Healthcare Inc. USA). A specialist physician was responsible for monitoring ECG and blood pressure responses during the test and recording subject’s signs and symptoms throughout the test.

Biochemical assessments

Blood samples were collected in the morning between 7:00 and 9:00 am after an overnight fasting. Plasma glucose and non-esterified fatty acids (NEFA) were assessed by KONELAB 20XTi analyzer (Thermo Fischer Scientific inc.Waltham, MA, USA). Plasma insulin was assessed by chemiluminescent immunoassay using the IMMULITE analyzer (Diagnostic Products Corporation, Los Angeles). The intra- and inter-assay CVs were 3.4% and 2.0% for glucose, 11% and 3.4% for insulin, and 7.4% and 8.4% for NEFA, respectively. The HOMA-IR index (homeostatic model assessment of insulin resistance) was calculated as (fasting glucose x fasting insulin/22.5). Serum leptin was assessed using human leptin (ELISA; Diagnostic Systems Laboratories, Inc., Webster, TX). Total adiponectin was measured by an enzyme immunoassay method using the Quantikine human total adiponectin/Acrp30 immunoassay (R&D Systems, Minneapolis, MN). Inter- and intra-assay coefficients of variation (CVs) were 2.2% and 2.7% for leptin, 3.3% and 4.3% for adiponectin, respectively

Insulin sensitivity

Whole-body insulin sensitivity was determined by the 75-g oral glucose tolerance test (OGTT). The test was performed for a subset of subjects (n=24) and insulin sensitivity index (Matsuda index) was calculated using the 0, 60 min and 120 min time points according to Matsuda and DeFronzo [6].

Serum NMR spectroscopy

All serum samples were analyzed using a high-throughput serum NMR metabonomics platform; the experimental protocols including sample preparation and NMR spectroscopy have been described in detail elsewhere[7]. This methodology has recently been applied in various large-scale epidemiological and genetic studies[8,9]. The NMR metabolomics methodology provides comprehensive quantitative information on various amino acids,

glycolysis intermediates, fatty acid composition and degree of saturation and lipoprotein subclass distributions. Altogether 130 metabolites were assessed.

Subcutaneous adipose tissue biopsies

Twenty-four participants agreed to donate subcutaneous adipose tissue biopsies, which were obtained under local anesthesia after overnight fasting. A region 5 cm lateral from the umbilicus either to the left side or right side was sterilized. A small intracutaneous injection was made, and 2 ml of a local anesthetic agent (lidocaine) was injected. After 5 min, anesthesia was confirmed, skin was sterilized again and 16 G, 40 mm needle, was then adapted to a 50-mL syringe and 10ml of 0.9% sodium-chloride was aspirated. Approximately two-third of the length of the needle was inserted into the subcutaneous fat, and 5 ml of 0.9% sodium chloride was injected. The needle piston was then pulled back maximally and released until it was locked by a stopper, thereby creating a vacuum. Tissue resistance was created by gripping the abdominal skin with one hand while the other hand rotated the needle throughout the tissue in back and forth motion. Once the tissue was aspirated by the syringe, the needle was withdrawn, and the piston was removed. The adipose tissue samples were washed with saline solution, and were immediately frozen in liquid nitrogen and stored at -80°C.

Skeletal muscle biopsies

Twenty-four participants agreed to donate skeletal muscle biopsies, which were obtained under local anesthesia after overnight fasting. Biopsies were obtained from the vastus lateralis dx muscle with a 5-mm Bergström biopsy needle, midway between the patella and greater trochanter. A region and the optimum depth for muscle biopsy were confirmed by ultrasound imaging. The skin of the identified location was sterilized and 4 ml of local anesthetic agent (lidocaine) was injected in to the procedure area. A cooling pack was then

applied on the location. After 10 minutes, anesthesia was confirmed, skin was sterilized again and small stab incision was made with surgical scalpel. Subsequently, the biopsy needle attached to a syringe was introduced perpendicularly into the incision. The piston was then pulled back maximally creating a vacuum and sample was obtained. After the muscle biopsy was obtained, pressure was applied to the incision site for hemostasis. The muscle sample was cleaned of any visible connective and adipose tissue as well as blood and was frozen immediately in liquid nitrogen (−180°C) and stored at −80°C.

RNA extraction

Total RNA was extracted from biopsies using the FastPrep system (MP Biomedicals, France) and the RNeasy Lipid Tissue Mini Kit (QIAGEN, Gaithersburg, MD, USA) according to manufacturer's instructions. Total RNA was digested on column with the RNase-free DNase set (QIAGEN) during RNA isolation. The quality of the total RNA was studied using a 2100 Bioanalyzer (Agilent, Santa Clara, CA, USA) and Experion Automated Electrophoresis Station (BioRad, Hercules, CA, USA). The total RNA was amplified and processed using the Gene Chip 3´ IVT Express Kit (Affymetrix, Santa Clara, CA, USA) and hybridized on Affymetrix Human Genome U219 Array Plates. The samples of this study have been submitted to Array Express. Array Express data are fully accessible in E-MTAB-2649.

Microarray analysis

The total RNA was amplified and processed using the GeneChip 3´IVT Express Kit (Affymetrix, Santa Clara, CA, USA) and hybridized on Affymetrix Human Genome U219 Array Plates as described previously [10]. Microarray data was pre-processed by the Robust Multiarray Averaging (RMA) algorithm in the R package affy [11-13]. Differentially expressed genes (DEG) were identified with the limma R package utilizing linear modeling and

empirical Bayes methods. Raw p-values were adjusted using the Benjamini and Hochberg multiple adjustment method [14].

Gene enrichment analysis

The enriched Gene Ontology (GO) terms and Kyoto Encyclopedia of Genes and Genomes (KEGG) pathways for a given gene set were calculated using the R packages GOStats and KEGG.db. In the enrichment analysis, all human ENSEMBL genes were used as a background gene group and categories with a p-value lower than 0.05 were considered significantly enriched. Genes related to HOMA-IR were identified using the following two criteria: Genes were differentially expressed in our DEG – analysis between the low HOMA-IR and high HOMA-IR groups with adjusted p-value <0.05 or genes fold change >= 2 between the low and high HOMA-IR groups.

For the gene pathways derived from KEGG enrichment analysis, the mean-centroid value representing the “activity” of the regulated part of the pathway was computed by normalizing the expression levels of all subset genes to a mean of zero and a variance of 1 across all individuals. The mean centroids have previously been shown to correlate with various metabolic and physiologic parameters [15,16], and may therefore be used to assess gene expression patterns that are associated with metabolic diseases.

Protein extraction from skeletal muscle biopsies and Western blot analysis

Muscle biopsies were homogenized in ice-cold lysis buffer [20 mM Tris-HCl (pH 7.4), 1 mM EDTA, 150mM NaCl, 100 mM β-glycerophosphate, 1 mM Na3VO4, 1 mM DTT, 1% Triton-X-100], supplemented with protease and phosphatase inhibitors inhibitors (Sigma Aldrich, St Louis, MO, USA). Thirty to sixty micrograms of muscle lysate samples were separated by SDS-Page using 4-20% gradient gels on Criterion electrophoresis cell (Bio-Rad Laboratories,

Richmond, CA). Proteins were transferred to nitrocellulose membranes at 300-mA constant current on ice at 4ºC. Membranes were blocked in TBS containing 5% nonfat dry milk for 1 hour at room temperature (RT), and then probed overnight at 4ºC with primary antibodies purchased from Cell Signaling Technology (Danvers, MA, USA) (p-Akt, p-IRβ and p-AS160), Sigma-Aldrich (anti-GAPDH) and Abcam (MitoProfile® Total OXPHOS Rodent WB Antibody Cocktail, Abcam, Cambridge, MA, USA). All antibodies were diluted 1:1.000 (except anti-GAPDH (housekeeping, which was diluted 1:40.000) in TBS containing 5% nonfat dry milk. Membranes were then washed with TBS containing 0.1% Tween-20 (TBS-T) followed by 1 hour incubation with the secondary antibody. Odyssey anti-rabbit IRDye 800 and Odyssey anti-mouse IRDye 600 (LI-COR Biosciences, Lincoln, NE, USA) were used as a secondary antibody. Blots were visualized and quantified using Odyssey CLX Infrared Imager of Li-COR and manufacturer's software. When reprobing was needed, the membranes were incubated in 0.2 M NaOH for 10 min at RT, washed with TBS and reprobed with appropriate antibodies. All samples were run in the same gel to minimize the variability and the quantitative results for each protein were normalized to GAPDH.

References

1 Volgyi, E. *et al.* Effect of long-term leisure time physical activity on lean mass and fat mass in girls during adolescence. *J Appl Physiol (1985)* **110**, 1211-1218, doi:10.1152/japplphysiol.00996.2010 (2011).

2 Cheng, S. *et al.* Trait-specific tracking and determinants of body composition: a 7-year follow-up study of pubertal growth in girls. *BMC medicine* **7**, 5, doi:10.1186/1741-7015-7-5 (2009).

3 Ojanen, X. *et al.* Comparison of vertebral bone marrow fat assessed by 1H MRS and inphase and out-of-phase MRI among family members. *Osteoporosis international : a journal established as result of cooperation between the European Foundation for Osteoporosis and the National Osteoporosis Foundation of the USA* **25**, 653-662, doi:10.1007/s00198-013-2472-9 (2014).

4 Borra, R. J. *et al.* Nonalcoholic fatty liver disease: rapid evaluation of liver fat content with in-phase and out-of-phase MR imaging. *Radiology* **250**, 130-136, doi:10.1148/radiol.2501071934 (2009).

5 Furuyama, J. K. *et al.* A pilot validation of multi-echo based echo-planar correlated spectroscopic imaging in human calf muscles. *NMR in biomedicine* **27**, 1176-1183, doi:10.1002/nbm.3171 (2014).

6 Matsuda, M. & DeFronzo, R. A. Insulin sensitivity indices obtained from oral glucose tolerance testing: comparison with the euglycemic insulin clamp. *Diabetes care* **22**, 1462-1470 (1999).

7 Soininen, P. *et al.* High-throughput serum NMR metabonomics for cost-effective holistic studies on systemic metabolism. *The Analyst* **134**, 1781-1785, doi:10.1039/b910205a (2009).

8 Stancakova, A. *et al.* Effects of 34 risk loci for type 2 diabetes or hyperglycemia on lipoprotein subclasses and their composition in 6,580 nondiabetic Finnish men. *Diabetes* **60**, 1608-1616, doi:10.2337/db10-1655 (2011).

9 Wurtz, P. *et al.* Metabolic signatures of insulin resistance in 7,098 young adults. *Diabetes* **61**, 1372-1380, doi:10.2337/db11-1355 (2012).

10 Munukka, E. *et al.* Gut-adipose tissue axis in hepatic fat accumulation in humans. *Journal of hepatology* **61**, 132-138, doi:10.1016/j.jhep.2014.02.020 (2014).

11 Gautier, L., Cope, L., Bolstad, B. M. & Irizarry, R. A. affy--analysis of Affymetrix GeneChip data at the probe level. *Bioinformatics* **20**, 307-315, doi:10.1093/bioinformatics/btg405 (2004).

12 Bolstad, B. M., Irizarry, R. A., Astrand, M. & Speed, T. P. A comparison of normalization methods for high density oligonucleotide array data based on variance and bias. *Bioinformatics* **19**, 185-193 (2003).

13 Gentleman, R. C. *et al.* Bioconductor: open software development for computational biology and bioinformatics. *Genome biology* **5**, R80, doi:10.1186/gb-2004-5-10-r80 (2004).

14 Benjamini Y, H. Y. Controlling the False Discovery Rate: A Practical and Powerful Approach to Multiple Testing. *Journal of the Royal Statistical Society Series B* **57**, 289-300. (1995).

15 Mootha, V. K. *et al.* PGC-1alpha-responsive genes involved in oxidative phosphorylation are coordinately downregulated in human diabetes. *Nature genetics* **34**, 267-273, doi:10.1038/ng1180 (2003).

16 Pietilainen, K. H. *et al.* Global transcript profiles of fat in monozygotic twins discordant for BMI: pathways behind acquired obesity. *PLoS medicine* **5**, e51, doi:10.1371/journal.pmed.0050051 (2008).

Table S2. General characteristics and serum metabolites in normal weight individuals stratified by low and high HOMA-IR groups (MIXED model estimated marginal means with 95% confidence intervals are given taking into account genetic similarity and shared environment (daughter and mother) and contrast estimates' p-values were used to localize the significant differences between the two groups and group by generation interaction).

	Low HOMA-IR n=20		High HOMA-IR n=20			
	Mean	95% CI	Mean	95% CI	p-value	Group by generation
Age	35.6	(34.2, 37.1)	36.9	(35.3, 38.5)	0.245	0.192
Height (cm)	168.1	(165.4, 170.8)	166.0	(163.1, 168.8)	0.267	0.163
Weight (kg)	59.2	(56.3, 62.2)	58.8	(55.7, 61.9)	0.826	0.301
BMI (kg/height^2)	21.0	(20.2, 21.8)	21.3	(20.4, 22.1)	0.580	0.907
Percent body fat	23.5	(21.4, 25.2)	25.7	(23.7, 27.7)	0.471	0.927
FM (kg)	16.9	(14.8, 19.0)	21.2	(19.1, 23.3)	0.006	0.227
FFM (kg)	40.9	(39.5, 42.3)	40.5	(39.1, 42.0)	0.968	0.599
VAT (kg)	0.55	(0.47, 0.62)	0.60	(0.53, 0.67)	0.328	0.056
Liver fat (%)	2.5	(1.0, 4,0)	1.9	(0.4, 3.4)	0.440	0.766
HOMA-IR	0.9	(0.7, 1.2)	2.4	(2.1, 2.7)	<0.001	0.234
**Metabolites (mmol/l)**						
Betahydroxybutyrate	0.049	(0.028, 0.069)	0.071	(0.053, 0.089)	0.115	0.057
Acetate	0.040	(0.036, 0.043	0.041	(0.038, 0.045)	0.186	0.788
Acetoacetate	0.038	(0.031, 0.045)	0.035	(0.029, 0.042)	0.876	0.926
Alanine	0.380	(0.355, 0.405)	0.400	(0.377, 0.423)	0.218	0.494
Citrate	0.100	(0.093, 0.107)	0.104	(0.097, 0.111)	0.300	0.191
Creatinine	0.050	(0.045, 0.054)	0.051	(0.047, 0.055)	0.222	0.853
Glutamine	0.512	(0.492, 0.532)	0.540	(0.522, 0.557)	0.091	0.881
Glycerol	0.058	(0.048, 0.068)	0.069	(0.060, 0.078)	0.089	0.065
Glycine	0.256	(0.237, 0.276)	0.279	(0.261, 0.296)	0.508	0.486
Orosomucoid	1.221	(1.155, 1.288)	1.299	(1.238, 1.360)	0.026	0.845
Histidine	0.050	(0.040, 0.054)	0.056	(0.053, 0.059)	0.089	0.933
Isoleucine	0.035	(0.032, 0.045)	0.043	(0.038, 0.044)	0.010	0.598
Leucine	0.061	(0.056, 0.065)	0.067	(0.063, 0.071)	0.016	0.887
Valine	0.154	(0.145, 0.168)	0.170	(0.160, 0.181)	0.046	0.472
BCAA_sum	0.255	(0.236, 0.275	0.282	(0.262, 0.298)	0.029	0.714
Tyrosine	0.042	(0.038, 0.046)	0.046	(0.042, 0.049)	0.121	0.109
Phenylalanine	0.062	(0.058, 0.065)	0.064	(0.061, 0.067)	0.275	0.192
Pyruvate	0.067	(0.060, 0.075)	0.074	(0.067, 0.081)	0.147	0.703
Lactate	0.888	(0.796, 0.980)	0.936	(0.850, 1.022)	0.333	0.259
Urea	0.052	(0.045, 0.060)	0.050	(0.043, 0.057)	0.408	0.065

FM = fat mass; FFF = fat-free mass; VAT = visceral adipose tissue; HOMA-IR = homeostatic model assessment of insulin resistance.

Table S3. General characteristics of the participants with adipose tissue biopsies

	Low HOMA-IR n= 12		High HOMA-IR n=12			
	Mean	95% CI	Mean	95% CI	p-value	Group by generation
**Anthropometry**						
Age (years)	34.5	(31.8, 37.3)	36.5	(34.2, 38.9)	0.257	0.077
Height (cm)	162.4	(158.2, 166.6)	164.3	(160.7, 168.0)	0.484	0.033
Weight (kg)	59.4	(51.3, 67.6)	66.2	(59.1, 73.3)	0.201	0.966
BMI (kg/height(m)^2)	22.5	(19.8, 25.2)	24.5	(22.2, 28.7)	0.263	0.308
Percent body fat	34.8	(29.2, 40.4)	37.1	(30.8, 43,4)	0.567	0.189
**Body composition**						
FM (kg)	19.6	(13.0, 26.2)	24.7	(18.9, 30.5)	0.233	0.423
FFM (kg)	37.4	(34.8, 40.0)	38.6	(36.3, 40.8)	0.473	0.070
VAT (kg)	0.51	(0.34, 0.67)	0.67	(0.52, 0.81)	0.141	0.809
Liver fat (%)	4.3	(-5.6, 14.2)	4.9	(-2.1, 11.9)	0.912	0.714
**Metabolic biomarkers**						
Glucose (mmol/l)	5.3	(4.8, 5.8)	6.2	(5.7, 6.6)	0.011	0.112
Insulin (IU/l)	3.3	(-1.9, 6.9)	11.0	(7.9, 14.0)	0.003	0.372
HOMA-IR	0.8	(-0.4, 1.9)	3.1	(2.2, 4.1)	0.004	0.253
Matsuda index	155	(63, 248)	134	(63, 204)	0.694	0.162
**Lipids**						
FFA (mmol/l)	5.2	(4.1, 6.3)	3.8	(2.6, 4.8)	0.051	0.053
Triglyceride (mmol/l)	1.3	(0.8, 1.8)	1.1	(0.7, 1.6)	0.607	0.398
**Adipokines**						
Leptin (ng/ml)	18.2	(3.4, 33.0)	31.5	(18.8, 44.2)	0.168	0.443
Adiponectin (μg/ml)	12.9	(7.1, 18.7)	15.0	(9.9, 20.1)	0.571	0.834
**Diet**						
Energy (kcal)	1490	(1121, 1860)	1717	(1342, 2091)	0.374	0.235
Protein (E%)	17.7	(14.9, 20.7)	18.8	(15.8, 21.8)	0.600	0.796
Fat (E%)	36.5	(30.6, 42.5)	34.4	(28.4, 40.4)	0.604	0.304
Carbohydrates (E%)	45.8	(44.5, 49.6)	46.8	(45.6, 50.5)	0.574	0.227
**Aerobic fitness**						
LTPA (hours/week)	4.8	(3.4, 6.3)	4.0	(2.7, 5.2)	0.350	0.624
VO2max (ml/kg/min)	38.9	(32.8, 44.9)	37.7	(31.5, 43.8)	0.771	0.497

Data are given as mean ± SD. FM = fat mass; VAT = visceral adipose tissue; HOMA-IR = homeostatic model assessment of insulin resistance; Matsuda index = insulin sensitivity index; FFA = free fatty acids; E% = percentage of total energy intake.

Table S4. Differentially expressed genes in adipose tissue in high HOMA-IR subjects

Ensembl Name	HGNC Name	Entrez ID	logFC	AveExpr	adj.P.value
ENSG00000100985	MMP9	4318	-4.2126	5.6951	6.025622e-05
ENSG00000118785	SPP1	6696	-3.8442	6.0416	8.461089e-04
ENSG00000198759	EGFL6	25975	-3.7408	6.7637	2.684735e-02
ENSG00000133048	CHI3L1	1116	-3.3721	4.6608	3.753393e-04
ENSG00000095970	TREM2	54209	-3.3170	4.3825	1.376531e-04
ENSG00000090920	FCGBP	8857	-3.0492	5.0437	2.300321e-04
ENSG00000169442	CD52	1043	-2.9708	6.4737	8.461089e-04
ENSG00000216490	IFI30	10437	-2.8481	8.4997	6.680926e-05
ENSG00000102575	ACP5	54	-2.5980	4.8979	2.607116e-04
ENSG00000006074	CCL18	6362	-2.5969	5.0617	5.767516e-04
ENSG00000145107	TM4SF19	116211	-2.5404	3.9842	1.434831e-03
ENSG00000160255	ITGB2	3689	-2.4524	5.9790	1.407523e-04
ENSG00000090382	LYZ	4069	-2.2082	6.3177	1.502205e-02
ENSG00000136689	IL1RN	3557	-2.1624	3.8975	1.670117e-03
ENSG00000136167	LCP1	3936	-2.0942	6.8186	3.097486e-04
ENSG00000203747	FCGR3A	2214	-2.0019	5.0590	1.720502e-03
ENSG00000143110	C1orf162	128346	-1.9904	5.6576	2.826425e-05
ENSG00000133063	CHIT1	1118	-1.9436	4.3065	3.680905e-04
ENSG00000163563	MNDA	4332	-1.9389	5.0557	6.359734e-04
ENSG00000162511	LAPTM5	7805	-1.9170	7.6189	3.973101e-05
ENSG00000129226	CD68	968	-1.8556	6.2990	6.938508e-05
ENSG00000171860	C3AR1	719	-1.8519	7.2832	1.294469e-03
ENSG00000167851	CD300A	11314	-1.8374	5.0158	3.297507e-04
ENSG00000154277	GRAPL	400581	-1.8281	6.8833	1.565034e-02
ENSG00000154277	UCHL1	7345	-1.8281	6.8833	1.565034e-02
ENSG00000129277	CCL4	6351	-1.8140	4.3460	2.826425e-05
ENSG00000126264	HCST	10870	-1.8129	6.8779	6.120144e-05
ENSG00000038945	MSR1	4481	-1.8069	4.2170	6.680926e-05
ENSG00000011600	TYROBP	7305	-1.7935	8.5988	5.033554e-04
ENSG00000073737	DHRS9	10170	-1.7706	5.3240	8.280990e-03
ENSG00000172724	CCL19	6363	-1.7660	4.5959	1.045267e-04
ENSG00000019169	MARCO	8685	-1.7543	4.9737	1.391699e-02
ENSG00000132205	EMILIN2	84034	-1.7195	5.6311	1.236338e-04
ENSG00000143119	CD53	963	-1.7101	5.0301	7.595704e-03
ENSG00000163131	CTSS	1520	-1.6957	6.4091	1.984531e-03
ENSG00000140379	BCL2A1	597	-1.6701	3.6508	1.101920e-03
ENSG00000177575	CD163	9332	-1.6642	7.2344	8.223722e-03
ENSG00000115956	PLEK	5341	-1.6581	5.1257	8.676675e-04
ENSG00000123338	NCKAP1L	3071	-1.6486	5.0695	5.767516e-04
ENSG00000155659	VSIG4	11326	-1.6483	5.3933	3.361559e-03
ENSG00000000005	TNMD	64102	-1.6248	8.2510	3.568110e-03

ENSG00000149781	FERMT3	83706	-1.6099	4.6364	1.848497e-04
ENSG00000172927	MYEOV	26579	-1.6069	4.6216	4.112995e-03
ENSG00000158869	FCER1G	2207	-1.6065	7.5240	1.110010e-03
ENSG00000169508	GPR183	1880	-1.5951	4.0736	1.236971e-04
ENSG00000169413	RNASE6	6039	-1.5915	5.8083	5.161187e-03
ENSG00000108846	ABCC3	8714	-1.5883	3.8688	7.538656e-05
ENSG00000140678	ITGAX	3687	-1.5870	3.8380	2.564943e-04
ENSG00000106483	SFRP4	6424	-1.5827	5.8388	1.578617e-03
ENSG00000143226	FCGR2A	2212	-1.5823	5.6163	2.570696e-03
ENSG00000128340	RAC2	5880	-1.5430	4.2720	1.078249e-03
ENSG00000173369	C1QB	713	-1.5376	8.1014	6.770834e-03
ENSG00000159189	C1QC	714	-1.5061	6.3171	9.460348e-03
ENSG00000184060	ADAP2	55803	-1.5059	5.6590	5.092276e-04
ENSG00000148908	RGS10	6001	-1.5040	6.9394	2.300321e-04
ENSG00000100365	NCF4	4689	-1.4664	5.7870	5.460143e-04
ENSG00000139278	GLIPR1	11010	-1.4597	5.2272	3.421348e-03
ENSG00000000938	FGR	2268	-1.4327	4.4776	4.921802e-04
ENSG00000110446	SLC15A3	51296	-1.4326	6.9658	1.255367e-04
ENSG00000129116	PALLD	23022	-1.3952	7.9881	1.159016e-02
ENSG00000135838	NPL	80896	-1.3888	3.9259	1.520794e-03
ENSG00000102359	SRPX2	27286	-1.3878	8.0120	4.688438e-03
ENSG00000165025	SYK	6850	-1.3696	4.8599	1.407523e-04
ENSG00000106066	CPVL	54504	-1.3636	6.6336	8.657218e-03
ENSG00000023902	PLEKHO1	51177	-1.3577	6.3437	9.156239e-05
ENSG00000108691	CCL2	6347	-1.3572	5.8544	4.112995e-03
ENSG00000118640	VAMP8	8673	-1.3396	8.0310	6.680926e-05
ENSG00000155465	SLC7A7	9056	-1.3372	4.3553	2.920894e-03
ENSG00000102265	TIMP1	7076	-1.3234	5.8566	6.680926e-05
ENSG00000006747	SCIN	85477	-1.3210	4.3228	1.608570e-02
ENSG00000122694	GLIPR2	152007	-1.3069	7.6182	3.110614e-03
ENSG00000103490	PYCARD	29108	-1.3018	5.5755	6.680926e-05
ENSG00000103811	CTSH	1512	-1.3001	7.9881	8.148200e-03
ENSG00000121769	FABP3	2170	-1.2993	4.1873	2.160066e-03
ENSG00000176788	BASP1	10409	-1.2979	6.1234	1.003536e-03
ENSG00000116690	PRG4	10216	-1.2960	4.2521	3.298487e-02
ENSG00000170458	CD14	929	-1.2907	7.8462	9.034694e-03
ENSG00000161921	CXCL16	58191	-1.2799	4.2468	3.973101e-05
ENSG00000142669	SH3BGRL3	83442	-1.2734	7.2187	1.035707e-03
ENSG00000112149	CD83	9308	-1.2647	4.5331	4.496166e-04
ENSG00000181374	CCL13	6357	-1.2608	5.0927	5.627038e-03
ENSG00000174807	CD248	57124	-1.2429	8.6163	6.204111e-04
ENSG00000113389	NPR3	4883	-1.2412	5.1866	2.114564e-02
ENSG00000081237	PTPRC	5788	-1.2375	4.7871	1.433125e-03
ENSG00000173372	C1QA	712	-1.2221	7.5254	1.677977e-03

ENSG00000185862	EVI2B	2124	-1.2047	5.1911	7.069165e-03
ENSG00000100448	CTSG	1511	-1.2039	5.4088	2.655206e-02
ENSG00000131711	MAP1B	4131	-1.2001	7.8045	2.092482e-02
ENSG00000137976	DNASE2B	58511	-1.1904	3.7499	8.461089e-04
ENSG00000082074	FYB	2533	-1.1863	4.1619	6.865986e-04
ENSG00000111679	PTPN6	5777	-1.1784	4.9873	6.120144e-05
ENSG00000166926	MS4A6E	245802	-1.1670	3.5840	6.379363e-03
ENSG00000167261	DPEP2	64174	-1.1483	4.0710	6.680926e-05
ENSG00000145147	SLIT2	9353	-1.1477	6.3214	4.887578e-03
ENSG00000088827	SIGLEC1	6614	-1.1403	6.1234	2.745411e-03
ENSG00000163220	S100A9	6280	-1.1379	4.3108	4.565064e-02
ENSG00000137285	TUBB2B	347733	-1.1374	4.2968	5.767516e-04
ENSG00000100079	LGALS2	3957	-1.1351	3.6592	1.894860e-02
ENSG00000211899	IGHM	3507	-1.1326	3.7755	4.906514e-03
ENSG00000085265	FCN1	2219	-1.1286	3.9711	2.507055e-02
ENSG00000119681	LTBP2	4053	-1.1238	6.3793	2.222908e-02
ENSG00000155629	PIK3AP1	118788	-1.1065	4.3052	6.680926e-05
ENSG00000143153	ATP1B1	481	-1.0959	4.7097	1.032023e-03
ENSG00000165457	FOLR2	2350	-1.0914	7.0934	1.219098e-02
ENSG00000110077	MS4A6A	64231	-1.0876	7.5632	1.934047e-02
ENSG00000186517	ARHGAP30	257106	-1.0809	4.7390	6.325072e-04
ENSG00000155926	SLA	6503	-1.0636	4.5357	6.120144e-05
ENSG00000041982	TNC	3371	-1.0541	3.6269	7.142954e-03
ENSG00000129988	LBP	3929	-1.0492	4.0561	7.850479e-03
ENSG00000110079	MS4A4A	51338	-1.0488	5.3107	1.069320e-02
ENSG00000132465	IGJ	3512	-1.0426	4.1646	2.993693e-02
ENSG00000116701	NCF2	4688	-1.0422	4.4566	3.295742e-02
ENSG00000167553	TUBA1C	84790	-1.0387	8.5259	1.078249e-03
ENSG00000135596	MICAL1	64780	-1.0370	4.7214	1.646296e-03
ENSG00000196154	S100A4	6275	-1.0336	10.8375	4.779993e-03
ENSG00000095383	TBC1D2	55357	-1.0280	4.4114	1.438460e-04
ENSG00000095585	BLNK	29760	-1.0279	5.1833	2.241821e-04
ENSG00000120280	CXorf21	80231	-1.0258	3.6409	4.481579e-03
ENSG00000007264	MATK	4145	-1.0247	3.6066	8.280990e-03
ENSG00000137267	TUBB2A	7280	-1.0229	10.5952	1.443724e-02
ENSG00000051523	CYBA	1535	-1.0207	8.9309	5.767516e-04
ENSG00000149418	ST14	6768	-1.0149	3.6708	2.765706e-05
ENSG00000136250	AOAH	313	-1.0139	4.3063	2.191374e-03
ENSG00000154096	THY1	7070	-1.0097	6.3039	3.602488e-02
ENSG00000155761	SPAG17	200162	-1.0047	4.0315	7.552883e-03
ENSG00000147443	DOK2	9046	-1.0046	4.1498	1.273770e-04
ENSG00000154589	LY96	23643	-1.0035	6.5398	1.646296e-03
ENSG00000076716	GPC4	2239	-1.0027	5.6867	4.404691e-02
ENSG00000126860	EVI2A	2123	-1.0024	3.7921	8.741319e-04

ENSG00000168461	RAB31	11031	-0.9996	7.4467	1.319488e-03
ENSG00000110031	LPXN	9404	-0.9985	5.8157	5.190516e-03
ENSG00000182013	PNMAL1	55228	-0.9947	4.1345	3.553720e-02
ENSG00000101347	SAMHD1	25939	-0.9935	5.0067	2.191374e-03
ENSG00000155307	SAMSN1	64092	-0.9897	4.1924	1.666361e-02
ENSG00000101160	CTSZ	1522	-0.9880	8.8228	6.025622e-05
ENSG00000134202	GSTM3	2947	-0.9874	5.2589	1.247868e-02
ENSG00000124491	F13A1	2162	-0.9857	7.9950	1.219098e-02
ENSG00000160883	HK3	3101	-0.9843	3.8454	2.539542e-04
ENSG00000132965	ALOX5AP	241	-0.9762	6.7425	2.702214e-02
ENSG00000171729	TMEM51	55092	-0.9762	4.1682	6.182394e-04
ENSG00000142347	MYO1F	4542	-0.9744	3.9108	7.867816e-05
ENSG00000142227	EMP3	2014	-0.9730	5.0073	3.830491e-03
ENSG00000082781	ITGB5	3693	-0.9730	9.3211	3.753393e-04
ENSG00000042493	CAPG	822	-0.9701	5.6401	3.853054e-03
ENSG00000163823	CCR1	1230	-0.9618	3.6142	5.150506e-03
ENSG00000135929	CYP27A1	1593	-0.9615	7.1576	4.512025e-03
ENSG00000104774	MAN2B1	4125	-0.9588	6.3157	6.680926e-05
ENSG00000110719	TCIRG1	10312	-0.9579	5.8027	3.530718e-04
ENSG00000161570	CCL5	6352	-0.9547	4.6874	7.865451e-03
ENSG00000166825	ANPEP	290	-0.9530	4.8472	2.300321e-04
ENSG00000133027	PEMT	10400	-0.9475	8.2510	4.386508e-02
ENSG00000171051	FPR1	2357	-0.9450	4.2493	3.116875e-02
ENSG00000100364	C22orf9	23313	-0.9397	4.6707	6.120144e-05
ENSG00000170266	GLB1	2720	-0.9349	7.5362	4.591043e-03
ENSG00000171298	GAA	2548	-0.9343	6.0146	1.889026e-03
ENSG00000141480	ARRB2	409	-0.9318	4.0341	6.938508e-05
ENSG00000134668	SPOCD1	90853	-0.9289	3.6529	1.625706e-02
ENSG00000011422	PLAUR	5329	-0.9264	3.8824	2.516185e-03
ENSG00000142634	EFHD2	79180	-0.9262	4.4177	8.988641e-05
ENSG00000072694	FCGR2B	2213	-0.9260	6.9967	7.573369e-03
ENSG00000204634	TBC1D8	11138	-0.9252	6.9786	5.495883e-03
ENSG00000114405	C3orf14	57415	-0.9220	5.4218	2.198449e-03
ENSG00000174370	C11orf45	219833	-0.9205	3.7110	6.382403e-05
ENSG00000019582	CD74	972	-0.9204	7.9537	4.905768e-03
ENSG00000095319	NUP188	23511	-0.9091	6.1110	3.711332e-03
ENSG00000065600	TMEM206	55248	-0.9056	4.1368	2.817956e-04
ENSG00000006075	CCL3	6348	-0.9046	3.3403	3.098755e-02
ENSG00000164733	CTSB	1508	-0.8993	10.1744	1.183533e-04
ENSG00000123146	CD97	976	-0.8927	5.7007	1.677977e-03
ENSG00000171700	RGS19	10287	-0.8834	7.1603	6.888164e-04
ENSG00000170017	ALCAM	214	-0.8826	3.3946	1.123714e-02
ENSG00000140030	GPR65	8477	-0.8768	4.1066	1.918302e-03
ENSG00000187474	FPR3	2359	-0.8766	3.6571	1.289519e-02

ENSG00000139410	SDSL	113675	-0.8764	4.3813	5.647845e-03
ENSG00000104783	KCNN4	3783	-0.8762	3.7987	6.680926e-05
ENSG00000149256	ODZ4	26011	-0.8740	4.5985	6.978533e-03
ENSG00000198951	NAGA	4668	-0.8725	6.0153	3.181184e-03
ENSG00000198855	FICD	11153	-0.8683	5.1578	4.361889e-02
ENSG00000101336	HCK	3055	-0.8659	3.9093	5.082814e-03
ENSG00000100600	LGMN	5641	-0.8642	9.0924	2.043570e-02
ENSG00000168140	VASN	114990	-0.8626	5.7447	2.280105e-02
ENSG00000006327	TNFRSF12A	51330	-0.8621	4.2133	1.454460e-02
ENSG00000089327	FXYD5	53827	-0.8621	6.0189	4.908597e-03
ENSG00000159403	C1R	715	-0.8591	9.1072	6.009359e-03
ENSG00000131042	LILRB2	10288	-0.8511	4.0463	5.616314e-03
ENSG00000196743	GM2A	2760	-0.8494	4.3928	1.283518e-04
ENSG00000167996	FTH1	2495	-0.8490	9.8856	9.034694e-03
ENSG00000136404	TM6SF1	53346	-0.8421	4.2742	2.608098e-03
ENSG00000132334	PTPRE	5791	-0.8281	4.0430	3.704694e-04
ENSG00000139974	SLC38A6	145389	-0.8250	5.9287	3.800781e-02
ENSG00000130592	LSP1	4046	-0.8221	4.8788	8.741319e-04
ENSG00000189159	HN1	51155	-0.8192	4.6836	2.920894e-03
ENSG00000182853	VMO1	284013	-0.8144	3.8715	1.670117e-03
ENSG00000121966	CXCR4	7852	-0.8135	4.1517	8.482757e-03
ENSG00000196924	FLNA	2316	-0.8065	6.5085	1.980661e-02
ENSG00000135124	P2RX4	5025	-0.8049	4.3345	1.278596e-03
ENSG00000092871	RFFL	117584	-0.8025	5.4853	1.454460e-02
ENSG00000038427	VCAN	1462	-0.8018	5.0074	9.557316e-03
ENSG00000140931	CMTM3	123920	-0.8002	6.7619	1.074201e-04
ENSG00000213654	GPSM3	63940	-0.7968	3.9459	2.817956e-04
ENSG00000100368	CSF2RB	1439	-0.7944	4.4162	7.538656e-05
ENSG00000174125	TLR1	7096	-0.7943	4.0615	9.034694e-03
ENSG00000087495	PHACTR3	116154	-0.7887	3.6457	2.740147e-02
ENSG00000134516	DOCK2	1794	-0.7886	3.8155	3.973101e-05
ENSG00000145416	MARCH1	55016	-0.7867	3.7830	1.183533e-04
ENSG00000030582	GRN	2896	-0.7864	7.4538	3.020402e-03
ENSG00000128228	SDF2L1	23753	-0.7857	5.6948	9.362047e-03
ENSG00000178860	MSC	9242	-0.7791	3.8542	5.240788e-03
ENSG00000136235	GPNMB	10457	-0.7781	7.4751	1.552194e-02
ENSG00000204103	MAFB	9935	-0.7738	7.6903	1.640303e-02
ENSG00000151929	BAG3	9531	-0.7709	8.1654	2.524701e-02
ENSG00000101265	RASSF2	9770	-0.7708	4.3923	2.241821e-04
ENSG00000181195	PENK	5179	-0.7706	3.6113	3.389602e-02
ENSG00000184216	IRAK1	3654	-0.7663	6.5553	1.159146e-02
ENSG00000168546	GFRA2	2675	-0.7646	4.3019	8.666036e-03
ENSG00000102879	CORO1A	11151	-0.7623	4.0010	1.779112e-03
ENSG00000111348	ARHGDIB	397	-0.7599	8.1345	3.680905e-04

ENSG00000175063	UBE2C	11065	-0.7584	3.4212	2.560135e-02
ENSG00000084636	COL16A1	1307	-0.7579	6.2096	1.635653e-02
ENSG00000126246	TMEM149	79713	-0.7562	5.3747	2.766021e-02
ENSG00000145703	IQGAP2	10788	-0.7557	3.9675	4.418731e-03
ENSG00000142197	DOPEY2	9980	-0.7552	4.3771	1.144215e-03
ENSG00000187164	KIAA1598	57698	-0.7517	3.8452	1.556325e-03
ENSG00000117091	CD48	962	-0.7481	3.8612	4.906514e-03
ENSG00000104951	IL4I1	259307	-0.7474	3.5005	3.421348e-03
ENSG00000013364	MVP	9961	-0.7456	4.9228	1.434831e-03
ENSG00000133321	RARRES3	5920	-0.7450	5.8705	5.659261e-03
ENSG00000120708	TGFBI	7045	-0.7441	7.4011	4.659105e-02
ENSG00000091986	CCDC80	151887	-0.7435	8.7023	9.539514e-03
ENSG00000163154	TNFAIP8L2	79626	-0.7395	4.1865	2.752054e-03
ENSG00000142173	COL6A2	1292	-0.7365	5.9794	3.592493e-03
ENSG00000197535	MYO5A	4644	-0.7354	5.6396	4.641161e-04
ENSG00000162745	OLFML2B	25903	-0.7347	4.7562	1.076704e-03
ENSG00000026508	CD44	960	-0.7344	5.5764	1.918302e-03
ENSG00000163106	HPGDS	27306	-0.7335	4.3865	1.963330e-02
ENSG00000123243	ITIH5	80760	-0.7322	8.4928	6.995125e-03
ENSG00000164611	PTTG1	9232	-0.7316	3.5852	4.402233e-03
ENSG00000005059	CCDC109B	55013	-0.7288	4.3716	1.503215e-03
ENSG00000147324	MFHAS1	9258	-0.7260	4.2258	6.680926e-05
ENSG00000145649	GZMA	3001	-0.7244	5.5396	3.920885e-02
ENSG00000164877	MICALL2	79778	-0.7241	4.8180	3.097486e-04
ENSG00000063660	GPC1	2817	-0.7232	4.6609	8.770877e-04
ENSG00000146386	C6orf115	58527	-0.7222	5.1621	3.956396e-02
ENSG00000118971	CCND2	894	-0.7205	5.4461	3.654686e-02
ENSG00000188060	RAB42	115273	-0.7176	4.2016	7.210121e-03
ENSG00000146409	C6orf192	116843	-0.7160	6.5148	3.747989e-04
ENSG00000102524	TNFSF13B	10673	-0.7158	5.2289	7.583792e-04
ENSG00000166557	TMED3	23423	-0.7137	4.3405	1.371156e-04
ENSG00000198937	C6orf129	154467	-0.7134	5.5334	8.666036e-03
ENSG00000105223	PLD3	23646	-0.7121	4.4931	1.871937e-03
ENSG00000064763	FAR2	55711	-0.7097	4.2263	4.386508e-02
ENSG00000168995	SIGLEC7	27036	-0.7070	3.5169	4.707552e-05
ENSG00000123975	CKS2	1164	-0.7052	4.1422	4.571258e-02
ENSG00000134061	CD180	4064	-0.7045	3.7670	7.452339e-04
ENSG00000157456	CCNB2	9133	-0.7030	3.4393	4.857037e-02
ENSG00000114013	CD86	942	-0.7012	3.8173	2.241821e-04
ENSG00000141506	PIK3R5	23533	-0.6995	3.7025	4.496166e-04
ENSG00000109743	BST1	683	-0.6957	5.7497	1.966137e-02
ENSG00000187554	TLR5	7100	-0.6947	4.9057	1.832123e-02
ENSG00000130830	MPP1	4354	-0.6937	5.8477	6.896709e-04
ENSG00000134247	PTGFRN	5738	-0.6926	4.3864	5.903774e-03

ENSG00000184347	SLIT3	6586	-0.6894	6.9254	3.813241e-02
ENSG00000141526	SLC16A3	9123	-0.6870	3.8526	3.973101e-05
ENSG00000124126	PREX1	57580	-0.6849	4.1277	2.608098e-03
ENSG00000182197	EXT1	2131	-0.6809	6.8623	7.069165e-03
ENSG00000118503	TNFAIP3	7128	-0.6772	5.9733	5.150506e-03
ENSG00000115355	CCDC88A	55704	-0.6747	4.8841	1.135909e-02
ENSG00000158163	DZIP1L	199221	-0.6742	4.1354	3.157856e-02
ENSG00000110347	MMP12	4321	-0.6725	3.5920	5.352111e-03
ENSG00000115919	KYNU	8942	-0.6655	3.4951	1.725369e-02
ENSG00000107438	PDLIM1	9124	-0.6653	8.0924	2.191374e-03
ENSG00000213064	SFT2D2	375035	-0.6642	4.4394	8.332492e-03
ENSG00000141968	VAV1	7409	-0.6611	3.8173	6.382403e-05
ENSG00000010327	STAB1	23166	-0.6608	5.8306	6.245284e-03
ENSG00000105383	CD33	945	-0.6596	4.5548	1.955438e-02
ENSG00000171310	CHST11	50515	-0.6513	3.4123	8.676675e-04
ENSG00000085514	PILRA	29992	-0.6503	3.7378	8.220848e-05
ENSG00000102962	CCL22	6367	-0.6501	3.8261	4.538862e-03
ENSG00000131747	TOP2A	7153	-0.6500	3.4310	1.106748e-02
ENSG00000114541	FRMD4B	23150	-0.6482	4.1353	9.444753e-03
ENSG00000180340	FZD2	2535	-0.6442	3.7681	1.779112e-03
ENSG00000105559	PLEKHA4	57664	-0.6409	4.6771	3.680905e-04
ENSG00000139289	PHLDA1	22822	-0.6407	3.5315	1.550512e-02
ENSG00000135905	DOCK10	55619	-0.6392	4.3261	3.976493e-02
ENSG00000103222	ABCC1	4363	-0.6364	5.1016	1.376531e-04
ENSG00000075624	ACTB	60	-0.6354	8.6726	3.111005e-02
ENSG00000182463	TSHZ2	128553	-0.6347	6.6079	2.114564e-02
ENSG00000182372	CLN8	2055	-0.6339	4.4191	4.584038e-03
ENSG00000006042	TMEM98	26022	-0.6322	5.7004	1.988765e-02
ENSG00000060982	BCAT1	586	-0.6315	3.4439	4.055580e-04
ENSG00000140749	IGSF6	10261	-0.6309	3.6658	7.887870e-03
ENSG00000117676	RPS6KA1	6195	-0.6304	3.6224	4.496166e-04
ENSG00000137673	MMP7	4316	-0.6296	3.3144	4.584038e-03
ENSG00000049860	HEXB	3074	-0.6293	9.4162	6.680926e-05
ENSG00000148468	FAM171A1	221061	-0.6282	5.4422	9.967858e-03
ENSG00000120885	CLU	1191	-0.6233	4.4415	6.021482e-03
ENSG00000139178	C1RL	51279	-0.6208	7.2256	1.688518e-02
ENSG00000135094	SDS	10993	-0.6198	3.5268	3.727256e-03
ENSG00000051128	HOMER3	9454	-0.6180	4.4535	1.670117e-03
ENSG00000158710	TAGLN2	8407	-0.6173	6.6125	1.946357e-02
ENSG00000117410	ATP6V0B	533	-0.6173	7.2174	6.865986e-04
ENSG00000128815	WDFY4	57705	-0.6171	3.5251	2.235196e-03
ENSG00000138061	CYP1B1	1545	-0.6140	4.8004	3.593250e-02
ENSG00000211772	TRBC2	28638	-0.6136	3.8193	2.734829e-03
ENSG00000173110	HSPA6	3310	-0.6116	3.4160	1.407523e-04

ENSG00000173110	HSPA7	3311	-0.6116	3.4160	1.407523e-04
ENSG00000066294	CD84	8832	-0.6115	3.6897	6.598825e-03
ENSG00000184408	KCND2	3751	-0.6039	4.1940	3.186936e-02
ENSG00000023445	BIRC3	330	-0.6028	3.6713	4.170402e-04
ENSG00000179163	FUCA1	2517	-0.6014	6.7208	3.076386e-02
ENSG00000053918	KCNQ1	3784	-0.6009	4.2293	1.670117e-03
ENSG00000033867	SLC4A7	9497	-0.5936	5.9984	1.110010e-03
ENSG00000144959	NCEH1	57552	-0.5920	3.3924	5.901755e-03
ENSG00000145685	LHFPL2	10184	-0.5918	3.7072	5.540053e-03
ENSG00000145569	FAM105A	54491	-0.5913	4.2517	8.223722e-03
ENSG00000102393	GLA	2717	-0.5901	6.1653	7.452339e-04
ENSG00000177675	CD163L1	283316	-0.5900	3.6302	2.560135e-02
ENSG00000034152	MAP2K3	5606	-0.5879	5.6996	3.208530e-02
ENSG00000075884	ARHGAP15	55843	-0.5851	5.1098	4.719917e-02
ENSG00000118855	MFSD1	64747	-0.5841	7.0311	3.023184e-02
ENSG00000185803	GPR172A	79581	-0.5828	4.1695	1.434831e-03
ENSG00000090674	MCOLN1	57192	-0.5743	6.6215	7.498470e-03
ENSG00000135919	SERPINE2	5270	-0.5704	4.1012	9.557316e-03
ENSG00000163694	RBM47	54502	-0.5677	4.6340	9.034694e-03
ENSG00000166002	C11orf75	56935	-0.5677	4.7371	1.635653e-02
ENSG00000128245	YWHAH	7533	-0.5659	9.1465	7.223799e-03
ENSG00000136830	FAM129B	64855	-0.5650	6.6191	4.484059e-03
ENSG00000074800	ENO1	2023	-0.5619	10.6436	1.869533e-02
ENSG00000197746	PSAP	5660	-0.5616	11.0692	3.973101e-05
ENSG00000176273	TMEM20	159371	-0.5573	4.0240	2.345355e-02
ENSG00000177105	RHOG	391	-0.5559	5.6874	2.200018e-02
ENSG00000087586	AURKA	6790	-0.5552	3.9247	4.411230e-02
ENSG00000142156	COL6A1	1291	-0.5540	8.1056	2.797442e-03
ENSG00000169245	CXCL10	3627	-0.5538	3.9389	2.794897e-02
ENSG00000119408	NEK6	10783	-0.5523	5.2950	1.930056e-03
ENSG00000111799	COL12A1	1303	-0.5522	4.9824	1.544391e-02
ENSG00000159840	ZYX	7791	-0.5518	4.1699	7.329702e-04
ENSG00000187688	TRPV2	51393	-0.5514	3.4706	4.496166e-04
ENSG00000188820	FAM26F	441168	-0.5495	4.3937	1.335997e-03
ENSG00000130816	DNMT1	1786	-0.5419	6.1854	2.767834e-03
ENSG00000196639	HRH1	3269	-0.5411	4.1817	1.720590e-02
ENSG00000168994	C6orf145	221749	-0.5407	8.3577	1.632061e-02
ENSG00000103569	AQP9	366	-0.5390	3.5047	3.973101e-05
ENSG00000121552	CSTA	1475	-0.5389	8.9171	3.654686e-02
ENSG00000101335	MYL9	10398	-0.5380	9.1017	3.706469e-02
ENSG00000137491	SLCO2B1	11309	-0.5373	3.7249	9.569730e-03
ENSG00000121152	NCAPH	23397	-0.5359	3.5933	3.200586e-02
ENSG00000185905	C16orf54	283897	-0.5344	3.4380	2.765706e-05
ENSG00000150681	RGS18	64407	-0.5335	3.6244	2.560135e-02

ENSG00000101916	TLR8	51311	-0.5303	3.4039	7.249252e-03
ENSG00000140450	ARRDC4	91947	-0.5297	4.1244	5.507830e-04
ENSG00000119655	NPC2	10577	-0.5295	10.1503	5.033554e-04
ENSG00000112773	FAM46A	55603	-0.5292	4.9212	5.043627e-03
ENSG00000162734	PEA15	8682	-0.5281	8.5779	7.109835e-03
ENSG00000059378	PARP12	64761	-0.5280	6.6812	1.490908e-02
ENSG00000166839	ANKDD1A	348094	-0.5274	3.6244	3.269385e-02
ENSG00000165704	HPRT1	3251	-0.5273	6.4164	9.421601e-03
ENSG00000138071	ACTR2	10097	-0.5271	6.4128	1.696812e-02
ENSG00000072110	ACTN1	87	-0.5266	7.5101	4.659105e-02
ENSG00000196878	LAMB3	3914	-0.5262	4.8617	2.276328e-02
ENSG00000172817	CYP7B1	9420	-0.5256	3.8026	4.508849e-02
ENSG00000196664	TLR7	51284	-0.5249	3.3965	1.677977e-03
ENSG00000105967	TFEC	22797	-0.5223	3.4676	1.688366e-03
ENSG00000176845	METRNL	284207	-0.5216	7.1978	4.438985e-03
ENSG00000130775	C1orf38	9473	-0.5210	3.7305	4.436236e-03
ENSG00000153395	LPCAT1	79888	-0.5204	6.0647	2.763320e-02
ENSG00000067057	PFKP	5214	-0.5203	4.8659	1.201837e-02
ENSG00000146232	NFKBIE	4794	-0.5201	4.2664	8.999611e-03
ENSG00000015475	BID	637	-0.5198	5.8057	8.148200e-03
ENSG00000143891	GALM	130589	-0.5181	5.2875	2.056795e-02
ENSG00000135842	FAM129A	116496	-0.5174	5.5697	4.055580e-04
ENSG00000037897	METTL1	4234	-0.5173	4.8886	4.938753e-02
ENSG00000112343	TRIM38	10475	-0.5147	6.2754	7.202342e-03
ENSG00000074410	CA12	771	-0.5113	4.0584	1.335095e-02
ENSG00000110324	IL10RA	3587	-0.5094	3.4248	4.745159e-03
ENSG00000123342	MMP19	4327	-0.5085	4.4444	1.799636e-02
ENSG00000136048	DRAM1	55332	-0.5067	3.9543	6.833061e-04
ENSG00000131238	PPT1	5538	-0.5051	6.5989	4.082604e-02
ENSG00000105619	TFPT	29844	-0.5019	5.4394	1.159146e-02
ENSG00000023892	DEF6	50619	-0.5018	3.3987	1.646296e-03
ENSG00000097021	ACOT7	11332	-0.5017	5.6216	4.064538e-02
ENSG00000065308	TRAM2	9697	-0.5006	5.4944	6.024635e-03
ENSG00000110906	KCTD10	83892	-0.5003	6.4378	2.350022e-02
ENSG00000147416	ATP6V1B2	526	-0.4996	6.3336	4.242012e-02
ENSG00000182326	C1S	716	-0.4995	6.0837	4.140315e-03
ENSG00000182578	CSF1R	1436	-0.4983	3.9343	2.391077e-03
ENSG00000177239	MAN1B1	11253	-0.4982	5.6817	9.930822e-03
ENSG00000153012	LGI2	55203	-0.4982	3.3858	7.109835e-03
ENSG00000175352	NRIP3	56675	-0.4979	3.3179	9.539514e-03
ENSG00000071553	ATP6AP1	537	-0.4969	6.6027	1.635653e-02
ENSG00000104870	FCGRT	2217	-0.4968	9.0520	5.595187e-04
ENSG00000118705	RPN2	6185	-0.4955	9.7188	1.177431e-02
ENSG00000115756	HPCAL1	3241	-0.4947	4.9114	9.741337e-03

ENSG00000180891	CUEDC1	404093	-0.4938	3.8841	1.778295e-02
ENSG00000186469	GNG2	54331	-0.4937	4.4461	1.749120e-03
ENSG00000110911	SLC11A2	4891	-0.4905	4.9203	2.456512e-03
ENSG00000109113	RAB34	83871	-0.4902	6.8970	2.914909e-02
ENSG00000188042	ARL4C	10123	-0.4901	3.9953	2.634255e-03
ENSG00000133195	SLC39A11	201266	-0.4865	4.2844	3.910542e-02
ENSG00000240972	MIF	4282	-0.4859	9.5784	1.137927e-02
ENSG00000145335	SNCA	6622	-0.4840	4.0366	2.482605e-03
ENSG00000213977	TAX1BP3	30851	-0.4824	9.3152	1.946357e-02
ENSG00000124357	NAGK	55577	-0.4820	7.6685	1.395297e-02
ENSG00000184293	CLECL1	160365	-0.4815	3.4570	3.097486e-04
ENSG00000127124	HIVEP3	59269	-0.4805	4.5015	3.430490e-02
ENSG00000198246	SLC29A3	55315	-0.4775	4.2696	3.680905e-04
ENSG00000188921	PTPLAD2	401494	-0.4764	3.5629	1.377608e-04
ENSG00000092820	EZR	7430	-0.4763	4.1467	1.501837e-03
ENSG00000186818	LILRB4	11006	-0.4739	3.3673	5.811373e-04
ENSG00000155252	PI4K2A	55361	-0.4739	6.1122	7.176551e-03
ENSG00000196507	TCEAL3	85012	-0.4735	7.5469	4.524030e-02
ENSG00000070882	OSBPL3	26031	-0.4734	3.5843	3.217957e-04
ENSG00000205220	PSMB10	5699	-0.4734	7.8582	7.814078e-03
ENSG00000185811	IKZF1	10320	-0.4730	3.5531	1.255367e-04
ENSG00000155660	PDIA4	9601	-0.4720	7.5804	3.282123e-02
ENSG00000197798	FAM118B	79607	-0.4709	5.5010	3.813121e-02
ENSG00000138180	CEP55	55165	-0.4703	3.1606	1.776073e-02
ENSG00000166311	SMPD1	6609	-0.4700	6.4277	2.515037e-02
ENSG00000080845	DLGAP4	22839	-0.4698	6.0729	1.378339e-02
ENSG00000185896	LAMP1	3916	-0.4693	9.6875	8.461089e-04
ENSG00000156299	TIAM1	7074	-0.4688	3.8434	1.433749e-03
ENSG00000175048	ZDHHC14	79683	-0.4675	4.5811	4.591252e-02
ENSG00000113648	H2AFY	9555	-0.4664	9.4881	5.767516e-04
ENSG00000093010	COMT	1312	-0.4659	7.1739	7.498470e-03
ENSG00000178573	MAF	4094	-0.4652	7.0739	7.552883e-03
ENSG00000175664	C13orf26	122046	-0.4627	3.2393	9.967858e-03
ENSG00000059377	TBXAS1	6916	-0.4626	3.7387	1.004796e-03
ENSG00000113578	FGF1	2246	-0.4608	4.0664	2.453060e-02
ENSG00000165527	ARF6	382	-0.4593	7.2268	4.652109e-02
ENSG00000116786	PLEKHM2	23207	-0.4582	7.7962	1.160764e-02
ENSG00000104687	GSR	2936	-0.4579	5.0443	2.300321e-04
ENSG00000133805	AMPD3	272	-0.4564	3.5719	6.245284e-03
ENSG00000175356	SCUBE2	57758	-0.4535	7.7695	4.060058e-02
ENSG00000151327	FAM177A1	283635	-0.4529	7.1541	3.845665e-03
ENSG00000188483	IER5L	389792	-0.4514	3.8626	8.726906e-04
ENSG00000169403	PTAFR	5724	-0.4513	3.8538	6.767081e-03
ENSG00000093072	CECR1	51816	-0.4485	3.2407	7.865451e-03

ENSG00000106268	NUDT1	4521	-0.4482	6.9840	1.648399e-02
ENSG00000160014	CALM3	808	-0.4479	4.5089	1.434831e-03
ENSG00000234906	APOC2	344	-0.4470	3.2768	2.649331e-02
ENSG00000106105	GARS	2617	-0.4444	8.6859	3.648389e-02
ENSG00000110880	CORO1C	23603	-0.4428	6.4381	2.906068e-02
ENSG00000127334	DYRK2	8445	-0.4422	4.8202	1.408618e-02
ENSG00000176658	MYO1D	4642	-0.4416	3.7563	5.240788e-03
ENSG00000114770	ABCC5	10057	-0.4411	4.6747	1.048809e-02
ENSG00000028137	TNFRSF1B	7133	-0.4384	4.3181	1.159146e-02
ENSG00000099998	GGT5	2687	-0.4375	4.2548	2.791138e-02
ENSG00000181649	PHLDA2	7262	-0.4369	3.2957	7.142954e-03
ENSG00000198715	C1orf85	112770	-0.4362	6.2374	2.052925e-02
ENSG00000184009	ACTG1	71	-0.4348	11.3125	1.559434e-03
ENSG00000197405	C5AR1	728	-0.4335	3.4486	5.092276e-04
ENSG00000121281	ADCY7	113	-0.4333	3.8045	4.112995e-03
ENSG00000064961	HMG20B	10362	-0.4329	7.0194	8.280990e-03
ENSG00000148143	ZNF462	58499	-0.4317	5.9889	4.419821e-02
ENSG00000172531	PPP1CA	5499	-0.4308	7.6307	2.276328e-02
ENSG00000083857	FAT1	2195	-0.4306	9.7808	3.813241e-02
ENSG00000090863	GLG1	2734	-0.4296	7.8502	2.649331e-02
ENSG00000178789	CD300LB	124599	-0.4294	3.4583	1.395364e-04
ENSG00000033100	CHPF2	54480	-0.4285	4.0327	9.481779e-03
ENSG00000139354	GAS2L3	283431	-0.4265	4.1533	1.686519e-02
ENSG00000164112	TMEM155	132332	-0.4262	3.3360	4.055332e-02
ENSG00000102445	C13orf18	80183	-0.4260	3.5352	3.913702e-03
ENSG00000108639	SYNGR2	9144	-0.4252	7.3551	2.810727e-02
ENSG00000169919	GUSB	2990	-0.4251	8.7198	3.553720e-02
ENSG00000123213	NLN	57486	-0.4247	3.8711	2.546321e-02
ENSG00000026751	SLAMF7	57823	-0.4245	3.3226	8.427114e-04
ENSG00000079931	MOXD1	26002	-0.4238	3.4872	1.887420e-02
ENSG00000131446	MGAT1	4245	-0.4223	6.0288	4.584038e-03
ENSG00000113552	GNPDA1	10007	-0.4223	4.8646	4.171321e-04
ENSG00000181467	RAP2B	5912	-0.4222	4.4598	1.441148e-02
ENSG00000196136	SERPINA3	12	-0.4211	3.6960	4.300196e-03
ENSG00000122986	HVCN1	84329	-0.4195	4.1389	1.454460e-02
ENSG00000122359	ANXA11	311	-0.4189	9.5753	3.433284e-02
ENSG00000177885	GRB2	2885	-0.4152	7.8670	2.767834e-03
ENSG00000116260	QSOX1	5768	-0.4149	5.1628	1.698817e-02
ENSG00000158050	DUSP2	1844	-0.4142	3.4747	1.211096e-02
ENSG00000183943	PRKX	5613	-0.4116	4.2327	6.644722e-03
ENSG00000070961	ATP2B1	490	-0.4116	4.6044	1.439674e-02
ENSG00000107175	CREB3	10488	-0.4108	7.2239	3.826284e-02
ENSG00000197555	SIPA1L1	26037	-0.4107	4.5328	3.845935e-02
ENSG00000121297	TSHZ3	57616	-0.4074	4.1142	1.433522e-02

ENSG00000168496	FEN1	2237	-0.4060	4.3265	3.430490e-02
ENSG00000138760	SCARB2	950	-0.4059	8.2535	7.595704e-03
ENSG00000130755	GMFG	9535	-0.4058	8.0574	1.040839e-02
ENSG00000174600	CMKLR1	1240	-0.4023	3.8609	4.113835e-03
ENSG00000126883	NUP214	8021	-0.4019	5.1657	1.391699e-02
ENSG00000113088	GZMK	3003	-0.4011	3.7146	7.176551e-03
ENSG00000141574	SECTM1	6398	-0.3995	3.7122	3.813241e-02
ENSG00000087088	BAX	581	-0.3983	4.3705	1.529527e-03
ENSG00000157045	NTAN1	123803	-0.3978	6.8427	1.552194e-02
ENSG00000137161	CNPY3	10695	-0.3976	3.8696	3.239800e-02
ENSG00000171992	SYNPO	11346	-0.3962	3.8230	1.604016e-02
ENSG00000158714	SLAMF8	56833	-0.3931	3.6948	1.434831e-03
ENSG00000151176	PLBD2	196463	-0.3924	3.9030	4.001122e-02
ENSG00000042753	AP2S1	1175	-0.3916	8.2309	2.276328e-02
ENSG00000106366	SERPINE1	5054	-0.3907	3.5498	5.767516e-04
ENSG00000141655	TNFRSF11A	8792	-0.3907	3.3437	1.230336e-02
ENSG00000154493	C10orf90	118611	-0.3905	3.4791	3.048189e-02
ENSG00000156535	CD109	135228	-0.3900	4.0123	4.060058e-02
ENSG00000170909	OSCAR	126014	-0.3883	3.4854	1.559434e-03
ENSG00000123159	GIPC1	10755	-0.3869	5.9881	3.864027e-02
ENSG00000103426	CORO7	79585	-0.3857	3.7314	3.854490e-03
ENSG00000197629	MPEG1	219972	-0.3836	3.6884	4.055332e-02
ENSG00000166068	SPRED1	161742	-0.3831	5.9504	4.694557e-02
ENSG00000197461	PDGFA	5154	-0.3831	3.6543	3.704694e-04
ENSG00000160789	LMNA	4000	-0.3827	8.1736	4.753363e-02
ENSG00000225190	PLEKHM1	9842	-0.3818	7.2469	2.809048e-02
ENSG00000068724	TTC7A	57217	-0.3816	4.5471	1.686519e-02
ENSG00000168172	HOOK3	84376	-0.3796	3.7346	2.281770e-02
ENSG00000166501	PRKCB	5579	-0.3796	3.6233	9.967858e-03
ENSG00000095015	MAP3K1	4214	-0.3787	7.4689	1.556103e-02
ENSG00000074964	ARHGEF10L	55160	-0.3785	4.1962	2.625466e-03
ENSG00000130309	GLT25D1	79709	-0.3760	5.7498	1.239013e-02
ENSG00000147065	MSN	4478	-0.3755	8.9566	2.833560e-02
ENSG00000162711	NLRP3	114548	-0.3739	3.8593	1.255749e-03
ENSG00000167470	MIDN	90007	-0.3738	3.9255	5.693318e-03
ENSG00000143344	RGL1	23179	-0.3737	4.2395	3.547410e-02
ENSG00000058262	SEC61A1	29927	-0.3734	5.3521	4.350849e-02
ENSG00000076554	TPD52	7163	-0.3733	3.7816	3.104759e-02
ENSG00000090104	RGS1	5996	-0.3727	3.4022	1.502205e-02
ENSG00000100350	FOXRED2	80020	-0.3726	4.9647	3.750881e-02
ENSG00000112079	STK38	11329	-0.3714	7.7685	1.502205e-02
ENSG00000115902	SLC1A4	6509	-0.3697	3.8237	3.568110e-03
ENSG00000110090	CPT1A	1374	-0.3695	4.0328	3.430490e-02
ENSG00000198771	RCSD1	92241	-0.3691	3.7544	8.897199e-03

ENSG00000122122	SASH3	54440	-0.3679	3.4289	7.452339e-04
ENSG00000064601	CTSA	5476	-0.3679	8.2825	3.815903e-02
ENSG00000103066	PLA2G15	23659	-0.3661	3.5758	6.949938e-03
ENSG00000135077	HAVCR2	84868	-0.3659	3.5900	8.866022e-03
ENSG00000052749	RRP12	23223	-0.3651	3.8945	3.912868e-02
ENSG00000117724	CENPF	1063	-0.3633	3.3365	1.396341e-02
ENSG00000090339	ICAM1	3383	-0.3630	3.8747	1.935469e-03
ENSG00000100280	AP1B1	162	-0.3609	4.2082	1.925655e-02
ENSG00000183696	UPP1	7378	-0.3605	4.0371	6.987335e-03
ENSG00000168610	STAT3	6774	-0.3595	8.0915	7.367109e-03
ENSG00000119321	FKBP15	23307	-0.3584	4.5187	2.816589e-02
ENSG00000001036	FUCA2	2519	-0.3579	7.2354	3.910542e-02
ENSG00000087074	PPP1R15A	23645	-0.3576	4.2426	4.692469e-02
ENSG00000156471	PTDSS1	9791	-0.3567	6.4302	1.219098e-02
ENSG00000155366	RHOC	389	-0.3547	8.2140	4.350849e-02
ENSG00000162458	FBLIM1	54751	-0.3532	4.0887	7.849530e-03
ENSG00000011009	LYPLA2	11313	-0.3531	8.8756	4.125785e-02
ENSG00000173083	HPSE	10855	-0.3529	3.3220	1.139307e-03
ENSG00000125877	ITPA	3704	-0.3529	6.3840	4.746989e-02
ENSG00000136026	CKAP4	10970	-0.3506	7.4833	3.815903e-02
ENSG00000064652	SNX24	28966	-0.3504	4.0617	9.034694e-03
ENSG00000106785	TRIM14	9830	-0.3482	3.4734	1.434831e-03
ENSG00000142694	FAM176B	55194	-0.3476	4.4057	2.932830e-02
ENSG00000167900	TK1	7083	-0.3475	3.4520	3.693128e-02
ENSG00000156011	PSD3	23362	-0.3475	3.9434	3.020402e-03
ENSG00000139344	AMDHD1	144193	-0.3446	3.4535	1.137927e-02
ENSG00000140526	ABHD2	11057	-0.3441	3.9188	1.395297e-02
ENSG00000135269	TES	26136	-0.3413	4.9200	1.932962e-02
ENSG00000100092	SH3BP1	23616	-0.3412	4.4500	1.493366e-02
ENSG00000213658	LAT	27040	-0.3405	4.0965	4.147977e-02
ENSG00000119333	WDR34	89891	-0.3378	4.1186	4.633948e-03
ENSG00000135956	TMEM127	55654	-0.3358	4.9271	1.730190e-02
ENSG00000006534	ALDH3B1	221	-0.3348	3.9388	1.925655e-02
ENSG00000164002	DEM1	64789	-0.3343	3.8008	2.748678e-03
ENSG00000089041	P2RX7	5027	-0.3338	3.3507	1.371156e-04
ENSG00000130733	YIPF2	78992	-0.3324	5.6865	4.833957e-02
ENSG00000178927	C17orf62	79415	-0.3323	7.4500	2.627292e-02
ENSG00000160593	AMICA1	120425	-0.3321	3.3545	1.927249e-02
ENSG00000175274	TP53I11	9537	-0.3316	3.8453	2.932830e-02
ENSG00000122420	PTGFR	5737	-0.3313	3.4094	1.555092e-02
ENSG00000196576	PLXNB2	23654	-0.3311	5.8011	4.650457e-02
ENSG00000135090	TAOK3	51347	-0.3300	6.1567	4.361889e-02
ENSG00000173442	EHBP1L1	254102	-0.3289	4.6256	3.956396e-02
ENSG00000148344	PTGES	9536	-0.3268	4.0996	3.182118e-02

ENSG00000180353	HCLS1	3059	-0.3267	3.8553	3.811255e-02
ENSG00000088325	TPX2	22974	-0.3252	3.3510	4.639451e-02
ENSG00000102580	DNAJC3	5611	-0.3235	3.5224	4.327927e-02
ENSG00000244405	ETV5	2119	-0.3231	3.5049	3.696021e-02
ENSG00000184840	TMED9	54732	-0.3230	6.8110	7.586568e-03
ENSG00000162706	CADM3	57863	-0.3222	3.6953	2.906068e-02
ENSG00000161011	SQSTM1	8878	-0.3200	9.2101	3.811255e-02
ENSG00000105501	SIGLEC14	1E+08	-0.3195	3.3260	4.133234e-02
ENSG00000105501	SIGLEC5	8778	-0.3195	3.3260	4.133234e-02
ENSG00000074842	C19orf10	56005	-0.3185	7.5231	4.639451e-02
ENSG00000138744	NAAA	27163	-0.3184	4.1527	2.931448e-02
ENSG00000152291	TGOLN2	10618	-0.3177	6.5207	2.463280e-02
ENSG00000008283	CYB561	1534	-0.3175	4.0627	1.161896e-03
ENSG00000103966	EHD4	30844	-0.3175	4.7825	1.348472e-02
ENSG00000110876	SELPLG	6404	-0.3165	3.8729	1.643651e-02
ENSG00000109089	CDR2L	30850	-0.3164	3.8151	4.505244e-02
ENSG00000177628	GBA	2629	-0.3158	4.5539	4.361889e-02
ENSG00000108950	FAM20A	54757	-0.3149	3.6994	2.906068e-02
ENSG00000117632	STMN1	3925	-0.3144	4.0687	3.282123e-02
ENSG00000101109	STK4	6789	-0.3138	4.1359	2.184616e-02
ENSG00000122861	PLAU	5328	-0.3125	3.9291	3.767174e-02
ENSG00000105429	MEGF8	1954	-0.3109	4.9372	2.741618e-02
ENSG00000128645	HOXD1	3231	-0.3080	3.2456	3.517552e-02
ENSG00000143643	TTC13	79573	-0.3064	6.9945	2.868498e-02
ENSG00000122870	BICC1	80114	-0.3038	3.8262	3.157856e-02
ENSG00000175463	TBC1D10C	374403	-0.3036	3.5536	8.280990e-03
ENSG00000162723	SLAMF9	89886	-0.3023	3.6817	1.202145e-02
ENSG00000170425	ADORA2B	136	-0.3005	3.2655	4.678093e-02
ENSG00000205744	DENND1C	79958	-0.2983	3.7383	2.495517e-02
ENSG00000100122	CRYBB1	1414	-0.2982	3.3285	4.803143e-03
ENSG00000111252	SH2B3	10019	-0.2956	3.5755	1.247868e-02
ENSG00000147614	ATP6V0D2	245972	-0.2947	3.2432	1.749120e-03
ENSG00000134013	LOXL2	4017	-0.2939	3.7732	9.421601e-03
ENSG00000153560	UBP1	7342	-0.2926	6.7950	2.063782e-02
ENSG00000179630	C13orf31	144811	-0.2901	3.4206	2.222052e-02
ENSG00000162894	FAIM3	9214	-0.2878	3.2367	9.034694e-03
ENSG00000117009	KMO	8564	-0.2848	3.2013	1.396341e-02
ENSG00000128191	DGCR8	54487	-0.2840	3.8100	2.408286e-02
ENSG00000197694	SPTAN1	6709	-0.2836	3.8761	1.927249e-02
ENSG00000060558	GNA15	2769	-0.2833	3.3670	1.137635e-02
ENSG00000005844	ITGAL	3683	-0.2829	3.4588	3.156544e-02
ENSG00000122877	EGR2	1959	-0.2828	3.2672	1.329816e-02
ENSG00000184640	SEPT9	10801	-0.2807	3.6411	4.254679e-03
ENSG00000170312	CDK1	983	-0.2785	3.6335	4.811892e-02

ENSG00000123130	ACOT9	23597	-0.2777	6.5748	4.246917e-02
ENSG00000136731	UGGT1	56886	-0.2773	4.6726	4.652109e-02
ENSG00000006062	MAP3K14	9020	-0.2764	3.5147	4.350849e-02
ENSG00000177000	MTHFR	4524	-0.2757	5.1490	4.445021e-02
ENSG00000187955	COL14A1	7373	-0.2755	3.3408	3.186936e-02
ENSG00000152767	FARP1	10160	-0.2754	5.6466	1.472306e-02
ENSG00000109066	TMEM104	54868	-0.2741	3.8588	4.932405e-02
ENSG00000095303	PTGS1	5742	-0.2732	3.7089	1.985587e-02
ENSG00000155393	HEATR3	55027	-0.2722	3.4681	4.517999e-02
ENSG00000149177	PTPRJ	5795	-0.2720	3.2576	5.240788e-03
ENSG00000174371	EXO1	9156	-0.2703	3.2602	2.265105e-02
ENSG00000122254	HS3ST2	9956	-0.2690	3.3297	1.915782e-03
ENSG00000134955	SLC37A2	219855	-0.2689	3.3764	1.167172e-02
ENSG00000174238	PITPNA	5306	-0.2687	6.5414	1.455920e-02
ENSG00000164935	TM7SF4	81501	-0.2683	3.3183	1.642150e-03
ENSG00000160190	SLC37A1	54020	-0.2673	3.6681	3.387835e-02
ENSG00000084070	SMAP2	64744	-0.2670	3.6568	3.889199e-02
ENSG00000196950	SLC39A10	57181	-0.2667	3.5538	1.449689e-02
ENSG00000035862	TIMP2	7077	-0.2651	9.5579	3.490813e-02
ENSG00000135823	STX6	10228	-0.2650	5.1883	4.694557e-02
ENSG00000165752	STK32C	282974	-0.2646	3.8517	1.447449e-02
ENSG00000135709	KIAA0513	9764	-0.2637	3.9284	2.107862e-02
ENSG00000169499	PLEKHA2	59339	-0.2635	4.4709	1.049189e-02
ENSG00000185215	TNFAIP2	7127	-0.2610	3.5456	2.028922e-02
ENSG00000161671	C19orf63	284361	-0.2575	9.6865	4.363277e-02
ENSG00000113273	ARSB	411	-0.2572	3.4905	4.254679e-03
ENSG00000165617	DACT1	51339	-0.2538	3.4933	1.730190e-02
ENSG00000159202	UBE2Z	65264	-0.2534	5.8233	4.083666e-02
ENSG00000115935	WIPF1	7456	-0.2517	3.6918	3.620124e-02
ENSG00000174013	FBXO45	200933	-0.2502	4.7911	2.560135e-02
ENSG00000156273	BACH1	571	-0.2489	3.4708	3.697181e-02
ENSG00000187210	GCNT1	2650	-0.2478	3.4720	3.718935e-03
ENSG00000139629	GALNT6	11226	-0.2462	3.2674	1.498914e-02
ENSG00000105245	NUMBL	9253	-0.2460	3.7703	3.507791e-02
ENSG00000118257	NRP2	8828	-0.2454	3.9318	4.717011e-02
ENSG00000144843	ADPRH	141	-0.2450	3.5175	4.692469e-02
ENSG00000162522	KIAA1522	57648	-0.2446	3.6449	9.323076e-03
ENSG00000126882	FAM78A	286336	-0.2442	3.4573	1.840322e-02
ENSG00000041357	PSMA4	5685	-0.2418	9.2365	3.387835e-02
ENSG00000109943	CRTAM	56253	-0.2406	3.1782	9.539514e-03
ENSG00000149091	DGKZ	8525	-0.2402	7.9326	2.784478e-02
ENSG00000070190	DAPP1	27071	-0.2388	3.1802	1.887420e-02
ENSG00000089639	GMIP	51291	-0.2374	3.4107	1.552756e-02
ENSG00000171631	P2RY6	5031	-0.2370	3.3388	3.338680e-02

ENSG00000100767	PAPLN	89932	-0.2366	3.4485	1.455064e-02
ENSG00000101224	CDC25B	994	-0.2364	3.6981	1.455064e-02
ENSG00000158769	F11R	50848	-0.2356	5.4130	2.087358e-02
ENSG00000099814	KIAA0284	283638	-0.2341	3.9994	2.114564e-02
ENSG00000158882	TOMM40L	84134	-0.2265	3.7287	4.040204e-02
ENSG00000168077	SCARA3	51435	-0.2262	3.4472	3.150883e-02
ENSG00000197063	MAFG	4097	-0.2210	5.6046	4.621591e-02
ENSG00000172403	SYNPO2	171024	-0.2198	3.5332	1.877094e-02
ENSG00000167600	CYP2S1	29785	-0.2165	3.2829	2.791138e-02
ENSG00000002587	HS3ST1	9957	-0.2141	3.2970	2.284664e-02
ENSG00000180448	HMHA1	23526	-0.2122	3.5818	4.064538e-02
ENSG00000100055	CYTH4	27128	-0.2121	3.5445	2.265105e-02
ENSG00000128000	ZNF780B	163131	-0.2121	3.0625	4.551090e-02
ENSG00000141540	TTYH2	94015	-0.2118	3.5317	4.639451e-02
ENSG00000162302	RPS6KA4	8986	-0.2108	3.4935	2.906068e-02
ENSG00000139436	GIT2	9815	-0.2075	4.5393	3.811255e-02
ENSG00000132535	DLG4	1742	-0.2069	3.5004	2.375917e-02
ENSG00000178562	CD28	940	-0.2024	3.2221	3.864050e-02
ENSG00000187808	ANKRD58	347454	-0.2007	3.3000	4.462003e-02
ENSG00000078808	SDF4	51150	-0.2004	7.1049	4.112818e-02
ENSG00000008083	JARID2	3720	-0.1977	3.8150	1.025554e-02
ENSG00000114450	GNB4	59345	-0.1944	3.3302	1.454460e-02
ENSG00000157240	FZD1	8321	-0.1921	3.7054	3.662223e-02
ENSG00000115112	TFCP2L1	29842	-0.1727	3.3234	3.463339e-02
ENSG00000123643	SLC36A1	206358	-0.1720	3.4612	2.109735e-02
ENSG00000167394	ZNF668	79759	-0.1696	3.5389	4.386508e-02
ENSG00000133256	PDE6B	5158	-0.1692	3.2860	4.361889e-02
ENSG00000170581	STAT2	6773	-0.1692	4.0326	3.579518e-02
ENSG00000150867	PIP4K2A	5305	-0.1608	3.3320	5.156709e-03
ENSG00000089685	BIRC5	332	-0.1529	3.2316	3.665510e-02
ENSG00000172243	CLEC7A	64581	-0.1453	3.2296	3.116875e-02
ENSG00000136709	WDR33	55339	0.1820	4.0904	2.931448e-02
ENSG00000159921	GNE	10020	0.1838	3.7256	4.905237e-02
ENSG00000111011	RSRC2	65117	0.1885	8.0919	3.842603e-02
ENSG00000126091	ST3GAL3	6487	0.1917	3.6096	1.873013e-02
ENSG00000115109	EPB41L5	57669	0.2033	3.3473	3.620124e-02
ENSG00000138081	FBXO11	80204	0.2162	4.2127	1.447449e-02
ENSG00000143457	GOLPH3L	55204	0.2181	5.1118	4.601651e-02
ENSG00000213079	RBM16	22828	0.2184	7.0614	3.813241e-02
ENSG00000140153	WDR20	91833	0.2206	5.3592	2.276328e-02
ENSG00000109819	PPARGC1A	10891	0.2212	3.7075	3.579518e-02
ENSG00000144559	C3orf31	132001	0.2218	3.5749	3.009122e-02
ENSG00000055950	MRPL43	84545	0.2221	6.6043	4.571258e-02
ENSG00000165283	STOML2	30968	0.2265	7.7902	2.515082e-02

ENSG00000110344	UBE4A	9354	0.2271	5.3265	4.196178e-02
ENSG00000198721	PECI	10455	0.2278	6.7396	7.498417e-03
ENSG00000100823	APEX1	328	0.2290	9.7253	1.454460e-02
ENSG00000183571	PGPEP1L	145814	0.2314	3.1434	4.652109e-02
ENSG00000046647	GEMIN8	54960	0.2323	5.0423	4.366537e-02
ENSG00000165626	BEND7	222389	0.2335	3.7128	4.031827e-02
ENSG00000075142	SRI	6717	0.2345	7.5424	4.632482e-02
ENSG00000105819	PMPCB	9512	0.2360	3.7381	2.026366e-02
ENSG00000118181	RPS25	6230	0.2364	11.3974	3.168902e-02
ENSG00000187824	TMEM220	388335	0.2397	4.1999	2.043570e-02
ENSG00000121413	ZSCAN18	65982	0.2408	4.4379	2.222908e-02
ENSG00000137601	NEK1	4750	0.2423	4.6133	3.750881e-02
ENSG00000136986	DERL1	79139	0.2446	7.8411	4.184920e-02
ENSG00000153561	RMND5A	64795	0.2474	4.6500	1.454460e-02
ENSG00000118579	MED28	80306	0.2505	5.1126	4.060058e-02
ENSG00000175445	LPL	4023	0.2505	12.2388	4.115409e-02
ENSG00000135387	CAPRIN1	4076	0.2530	5.3958	2.482869e-02
ENSG00000168734	PKIG	11142	0.2543	6.2570	7.511043e-03
ENSG00000100372	SLC25A17	10478	0.2581	4.7779	4.448049e-02
ENSG00000065150	IPO5	3843	0.2603	8.5771	4.542764e-02
ENSG00000065548	ZC3H15	55854	0.2605	8.5589	3.468524e-02
ENSG00000143256	PFDN2	5202	0.2607	8.8638	3.430490e-02
ENSG00000102763	KIAA0564	23078	0.2613	5.8497	8.280990e-03
ENSG00000159346	ADIPOR1	51094	0.2617	6.2249	4.350849e-02
ENSG00000144724	PTPRG	5793	0.2643	4.7824	3.841218e-02
ENSG00000157502	MUM1L1	139221	0.2676	3.7360	3.978168e-02
ENSG00000085274	MYNN	55892	0.2681	4.9812	2.538318e-02
ENSG00000188938	FAM120AOS	158293	0.2686	5.4701	8.341510e-03
ENSG00000085788	DDHD2	23259	0.2702	6.7359	4.408800e-02
ENSG00000049245	VAMP3	9341	0.2727	8.5944	2.000965e-02
ENSG00000142676	RPL11	6135	0.2731	12.4992	3.191783e-02
ENSG00000162620	LRRIQ3	127255	0.2746	3.5174	4.386508e-02
ENSG00000181929	PRKAG1	5571	0.2748	7.6076	3.553720e-02
ENSG00000166260	COX11	1353	0.2763	6.6852	8.559185e-03
ENSG00000130024	PHF10	55274	0.2772	5.5691	9.597152e-03
ENSG00000128915	NARG2	79664	0.2787	5.2332	1.646296e-03
ENSG00000112983	BRD8	10902	0.2788	6.7406	1.987009e-02
ENSG00000186951	PPARA	5465	0.2794	3.9689	4.008728e-02
ENSG00000123636	BAZ2B	29994	0.2805	7.1324	2.990919e-02
ENSG00000167283	ATP5L	10632	0.2812	8.4290	4.542764e-02
ENSG00000166233	ARIH1	25820	0.2825	5.8630	2.791138e-02
ENSG00000116251	RPL22	6146	0.2839	10.6146	3.811255e-02
ENSG00000198742	SMURF1	57154	0.2847	3.8147	2.847136e-02
ENSG00000112339	HBS1L	10767	0.2860	3.9253	1.083439e-02

ENSG00000145741	BTF3	689	0.2862	10.3650	2.265105e-02
ENSG00000114416	FXR1	8087	0.2877	9.1055	4.688438e-03
ENSG00000125247	TMTC4	84899	0.2888	4.2056	3.748672e-02
ENSG00000129691	ASH2L	9070	0.2900	7.4383	3.706394e-02
ENSG00000105176	C19orf2	8725	0.2946	7.4451	1.625706e-02
ENSG00000163788	SNRK	54861	0.2950	6.6738	3.620124e-02
ENSG00000139684	ESD	2098	0.2951	10.4141	1.706472e-02
ENSG00000042317	SPATA7	55812	0.2955	3.9673	3.227593e-02
ENSG00000049883	PTCD2	79810	0.2971	3.6015	3.313962e-02
ENSG00000174233	ADCY6	112	0.2971	6.6369	1.945501e-02
ENSG00000180228	PRKRA	8575	0.2977	7.6632	3.750881e-02
ENSG00000180228	PRKRAP1	731716	0.2977	7.6632	3.750881e-02
ENSG00000104067	TJP1	7082	0.3001	8.3462	3.956396e-02
ENSG00000116906	GNPAT	8443	0.3008	7.8019	2.128215e-02
ENSG00000099954	CECR2	27443	0.3029	3.3904	3.543724e-02
ENSG00000167904	TMEM68	137695	0.3040	4.6257	2.052925e-02
ENSG00000113048	MRPS27	23107	0.3052	4.7245	2.560135e-02
ENSG00000158793	NIT1	4817	0.3054	5.7431	2.613134e-02
ENSG00000119820	YIPF4	84272	0.3064	5.8091	1.635653e-02
ENSG00000175575	PAAF1	80227	0.3065	7.3281	3.190879e-02
ENSG00000163512	AZI2	64343	0.3082	7.9263	6.299870e-03
ENSG00000146729	GBAS	2631	0.3087	8.7235	2.111676e-02
ENSG00000141325	CCDC45	90799	0.3097	5.0651	1.873975e-02
ENSG00000155313	USP25	29761	0.3102	6.8162	3.201490e-02
ENSG00000186106	ANKRD46	157567	0.3112	6.1533	5.190516e-03
ENSG00000182973	CNOT10	25904	0.3123	5.3973	4.387375e-02
ENSG00000145332	KLHL8	57563	0.3132	5.4042	2.580623e-02
ENSG00000166295	ANAPC16	119504	0.3143	9.2791	1.400120e-02
ENSG00000102910	LONP2	83752	0.3143	6.7142	1.825924e-02
ENSG00000168291	PDHB	5162	0.3162	9.3981	3.984008e-02
ENSG00000125901	MRPS26	64949	0.3167	7.8002	2.081134e-02
ENSG00000080822	CLDND1	56650	0.3174	8.0877	9.516731e-03
ENSG00000118418	HMGN3	9324	0.3209	8.1355	4.763868e-02
ENSG00000186205	MOSC1	64757	0.3252	10.5962	1.131379e-02
ENSG00000115947	ORC4L	5000	0.3272	6.1225	9.706928e-03
ENSG00000166455	C16orf46	123775	0.3293	3.5249	4.293568e-02
ENSG00000184752	NDUFA12	55967	0.3293	9.1976	8.741319e-04
ENSG00000101752	MIB1	57534	0.3297	5.1994	4.650457e-02
ENSG00000162623	TYW3	127253	0.3300	4.3166	5.693318e-03
ENSG00000182093	WRB	7485	0.3302	6.7075	4.125785e-02
ENSG00000102738	MRPS31	10240	0.3303	6.3055	3.811255e-02
ENSG00000117592	PRDX6	9588	0.3330	11.3942	8.897199e-03
ENSG00000143553	SNAPIN	23557	0.3343	9.3420	4.825860e-02
ENSG00000166902	MRPL16	54948	0.3344	8.8468	1.656257e-02

ENSG00000162735	PEX19	5824	0.3355	5.8163	3.702112e-03
ENSG00000100462	PRMT5	10419	0.3360	7.5552	3.543724e-02
ENSG00000075089	ACTR6	64431	0.3365	5.6452	2.871171e-02
ENSG00000119471	HSDL2	84263	0.3366	9.3265	1.329816e-02
ENSG00000164904	ALDH7A1	501	0.3377	5.2037	3.435490e-03
ENSG00000171490	RSL1D1	26156	0.3404	9.1126	4.115409e-02
ENSG00000169100	SLC25A6	293	0.3409	9.6433	2.931234e-02
ENSG00000155906	RMND1	55005	0.3412	4.9757	3.219258e-02
ENSG00000067177	PHKA1	5255	0.3440	4.1037	4.959310e-02
ENSG00000196704	AMZ2	51321	0.3444	8.8830	3.104759e-02
ENSG00000107537	PHYH	5264	0.3464	8.7979	1.391699e-02
ENSG00000129518	EAPP	55837	0.3482	7.3224	2.114564e-02
ENSG00000165410	CFL2	1073	0.3483	8.4376	6.644722e-03
ENSG00000106591	MRPL32	64983	0.3489	8.6801	3.582265e-02
ENSG00000157259	GATAD1	57798	0.3501	7.8473	2.265105e-02
ENSG00000136247	ZDHHC4	55146	0.3502	8.7567	2.484754e-02
ENSG00000185721	DRG1	4733	0.3506	9.3501	1.681502e-02
ENSG00000197006	METTL9	51108	0.3511	7.2420	1.205393e-02
ENSG00000132300	PTCD3	55037	0.3512	5.8274	5.150506e-03
ENSG00000096717	SIRT1	23411	0.3545	3.5765	1.400120e-02
ENSG00000164258	NDUFS4	4724	0.3545	9.9173	1.137635e-02
ENSG00000112304	ACOT13	55856	0.3547	8.3990	2.338210e-02
ENSG00000156162	DPY19L4	286148	0.3556	4.8763	3.910542e-02
ENSG00000049323	LTBP1	4052	0.3559	5.0280	4.703192e-02
ENSG00000069535	MAOB	4129	0.3561	7.9485	1.819997e-02
ENSG00000070761	C16orf80	29105	0.3572	7.6235	1.424345e-02
ENSG00000115317	HTRA2	27429	0.3591	6.1963	3.813241e-02
ENSG00000172292	LASS6	253782	0.3621	4.8858	3.622875e-02
ENSG00000198252	STYX	6815	0.3622	6.5944	4.887578e-03
ENSG00000165502	RPL36AL	6166	0.3648	9.1606	5.786997e-03
ENSG00000153914	SFRS12	140890	0.3665	5.2148	3.553720e-02
ENSG00000109184	DCUN1D4	23142	0.3665	6.4253	3.275181e-02
ENSG00000066027	PPP2R5A	5525	0.3695	8.9393	4.350849e-02
ENSG00000100722	ZC3H14	79882	0.3696	7.0280	2.702214e-02
ENSG00000010017	RANBP9	10048	0.3701	9.1054	1.801654e-02
ENSG00000070159	PTPN3	5774	0.3708	3.7607	4.848024e-03
ENSG00000116005	PCYOX1	51449	0.3709	8.3108	2.276628e-02
ENSG00000101365	IDH3B	3420	0.3719	7.9987	8.897199e-03
ENSG00000174100	MRPL45	84311	0.3731	7.6476	1.988765e-02
ENSG00000164056	SPRY1	10252	0.3758	8.1780	1.334235e-02
ENSG00000117625	RCOR3	55758	0.3758	5.5985	3.029993e-02
ENSG00000115084	SLC35F5	80255	0.3763	6.5518	4.008728e-02
ENSG00000143337	TOR1AIP1	26092	0.3793	7.3915	2.493966e-03
ENSG00000138175	ARL3	403	0.3805	8.1530	1.106345e-02

ENSG00000005801	ZNF195	7748	0.3807	6.0443	5.150506e-03
ENSG00000123607	TTC21B	79809	0.3810	5.0012	2.931448e-02
ENSG00000197147	LRRC8B	23507	0.3826	3.8520	4.445021e-02
ENSG00000138750	NUP54	53371	0.3836	7.9821	2.278166e-02
ENSG00000172340	SUCLG2	8801	0.3843	5.4303	8.149197e-03
ENSG00000163159	VPS72	6944	0.3844	7.5428	6.644722e-03
ENSG00000141076	CIRH1A	84916	0.3845	8.0260	3.468524e-02
ENSG00000154814	OXNAD1	92106	0.3856	5.1259	4.508849e-02
ENSG00000035928	RFC1	5981	0.3858	7.3045	9.539514e-03
ENSG00000108788	MLX	6945	0.3867	7.0644	1.144055e-02
ENSG00000178149	DALRD3	55152	0.3869	4.8420	3.570016e-02
ENSG00000146085	MUT	4594	0.3890	7.8740	4.879437e-03
ENSG00000137996	RTCD1	8634	0.3895	7.0128	3.915133e-02
ENSG00000133131	MORC4	79710	0.3925	4.7724	1.910213e-02
ENSG00000040341	STAU2	27067	0.3928	5.7316	9.930822e-03
ENSG00000168792	ABHD15	116236	0.3929	7.5811	2.810510e-02
ENSG00000175061	NCRNA00188	125144	0.3930	11.6045	8.148200e-03
ENSG00000162407	PPAP2B	8613	0.3946	9.4581	3.517552e-02
ENSG00000102349	KLF8	11279	0.3946	5.1760	3.940478e-02
ENSG00000104517	UBR5	51366	0.3956	7.7012	4.526152e-02
ENSG00000078399	HOXA10	3206	0.3981	5.6502	1.320940e-02
ENSG00000078399	HOXA9	3205	0.3981	5.6502	1.320940e-02
ENSG00000100209	HSCB	150274	0.3984	6.4319	2.816589e-02
ENSG00000119139	TJP2	9414	0.3987	8.1206	1.498914e-02
ENSG00000131051	RBM39	9584	0.3994	8.3544	2.056795e-02
ENSG00000134717	BTF3L4	91408	0.4005	7.9222	3.976493e-02
ENSG00000120256	LRP11	84918	0.4017	6.4042	2.969508e-02
ENSG00000148730	EIF4EBP2	1979	0.4023	8.2733	1.900821e-02
ENSG00000104343	UBE2W	55284	0.4035	7.0010	3.815903e-02
ENSG00000162496	DHRS3	9249	0.4064	6.7614	1.552194e-02
ENSG00000111275	ALDH2	217	0.4069	11.3352	7.552883e-03
ENSG00000155463	OXA1L	5018	0.4073	7.0895	1.749120e-03
ENSG00000177119	ANO6	196527	0.4077	8.1434	1.374302e-03
ENSG00000164008	C1orf50	79078	0.4078	7.8644	2.114564e-02
ENSG00000166352	C11orf74	119710	0.4100	6.9665	1.643651e-02
ENSG00000065243	PKN2	5586	0.4100	7.1480	3.590080e-02
ENSG00000151729	SLC25A4	291	0.4102	5.1898	3.111005e-02
ENSG00000033030	ZCCHC8	55596	0.4103	6.3504	4.194434e-02
ENSG00000169599	NFU1	27247	0.4107	8.4285	1.038245e-02
ENSG00000067248	DHX29	54505	0.4128	7.7440	1.356605e-02
ENSG00000166821	PEX11A	8800	0.4133	7.2373	4.650457e-02
ENSG00000151353	TMEM18	129787	0.4148	6.3887	3.337313e-03
ENSG00000008869	HEATR5B	54497	0.4151	7.7502	4.361889e-02
ENSG00000127452	FBXL12	54850	0.4169	7.3030	1.472691e-02

ENSG00000122484	RPAP2	79871	0.4188	5.0042	2.056795e-02
ENSG00000134419	RPS15A	6210	0.4194	9.6832	4.350849e-02
ENSG00000155542	C5orf35	133383	0.4225	4.3785	4.462003e-02
ENSG00000135972	MRPS9	64965	0.4232	7.8528	9.246164e-03
ENSG00000181191	PJA1	64219	0.4244	8.5119	2.238745e-02
ENSG00000081181	ARG2	384	0.4248	3.6203	3.842603e-02
ENSG00000104671	DCTN6	10671	0.4264	8.1807	3.811255e-02
ENSG00000198815	FOXJ3	22887	0.4295	8.5011	2.283633e-02
ENSG00000196531	NACA	4666	0.4299	11.7906	8.095342e-04
ENSG00000196531	NACA2	342538	0.4299	11.7906	8.095342e-04
ENSG00000196531	NACAP1	83955	0.4299	11.7906	8.095342e-04
ENSG00000144895	EIF2A	83939	0.4305	9.4938	1.829892e-02
ENSG00000152942	C4orf52	389203	0.4318	7.0486	4.848024e-03
ENSG00000152942	RAD17	5884	0.4318	7.0486	4.848024e-03
ENSG00000136158	SPRY2	10253	0.4353	7.5422	4.141315e-02
ENSG00000187325	TAF9B	51616	0.4358	4.6584	1.337370e-02
ENSG00000215021	PHB2	11331	0.4364	9.0995	6.598825e-03
ENSG00000116212	LRRC42	115353	0.4375	5.5745	3.547410e-02
ENSG00000151332	MBIP	51562	0.4399	7.2485	3.410269e-02
ENSG00000214517	PPME1	51400	0.4400	4.5401	1.648399e-02
ENSG00000180979	LRRC57	255252	0.4410	6.1493	1.772251e-02
ENSG00000131732	ZCCHC9	84240	0.4419	7.2812	4.913625e-02
ENSG00000096070	BRPF3	27154	0.4424	4.4207	2.275115e-02
ENSG00000175110	MRPS22	56945	0.4428	8.0440	5.190516e-03
ENSG00000101474	C20orf3	57136	0.4436	8.1898	4.075547e-02
ENSG00000131016	AKAP12	9590	0.4447	10.0325	3.978168e-02
ENSG00000090263	MRPS33	51650	0.4455	8.6586	9.220370e-03
ENSG00000056998	GYG2	8908	0.4457	7.3670	8.580707e-03
ENSG00000156735	BAG4	9530	0.4479	5.5013	4.373792e-02
ENSG00000079459	FDFT1	2222	0.4482	9.7120	1.422303e-02
ENSG00000155970	EFHA2	286097	0.4514	5.0229	1.840322e-02
ENSG00000141198	TOM1L1	10040	0.4532	4.4785	3.889199e-02
ENSG00000221909	FAM200A	221786	0.4534	4.5405	3.395086e-02
ENSG00000080561	MID2	11043	0.4548	5.7778	1.909074e-02
ENSG00000135336	ORC3L	23595	0.4562	6.6766	4.115409e-02
ENSG00000023516	AKAP11	11215	0.4567	8.4378	2.056795e-02
ENSG00000119689	DLST	1743	0.4585	4.6560	1.136916e-02
ENSG00000101126	ADNP	23394	0.4590	8.3841	3.453337e-02
ENSG00000115042	FAHD2A	51011	0.4601	5.3332	2.064702e-03
ENSG00000241399	CD302	9936	0.4609	9.6715	1.987389e-02
ENSG00000138764	CCNG2	901	0.4614	4.3123	2.265105e-02
ENSG00000163629	PTPN13	5783	0.4617	5.8695	4.012699e-02
ENSG00000153140	CETN3	1070	0.4620	6.9434	2.700059e-02
ENSG00000055211	C6orf72	116254	0.4632	8.8159	3.962170e-02

ENSG00000151748	SAV1	60485	0.4675	6.9198	2.442895e-02
ENSG00000115942	ORC2L	4999	0.4692	3.9836	1.721858e-02
ENSG00000116062	MSH6	2956	0.4699	4.8515	3.937548e-02
ENSG00000114784	EIF1B	10289	0.4752	8.4683	2.487472e-02
ENSG00000102038	SMARCA1	6594	0.4772	7.4725	3.813241e-02
ENSG00000196151	WDSUB1	151525	0.4774	6.1529	3.111005e-02
ENSG00000127995	CASD1	64921	0.4775	6.5541	4.461647e-02
ENSG00000178922	HYI	81888	0.4806	7.5465	2.265105e-02
ENSG00000181192	DHTKD1	55526	0.4818	7.4454	3.782419e-02
ENSG00000063046	EIF4B	1975	0.4833	8.1891	1.137635e-02
ENSG00000183513	C2orf64	493753	0.4836	7.1942	1.034524e-02
ENSG00000117118	SDHB	6390	0.4855	8.4649	8.280990e-03
ENSG00000054654	SYNE2	23224	0.4862	7.3689	4.300196e-03
ENSG00000067365	C16orf68	79091	0.4864	5.9219	3.649375e-02
ENSG00000112964	GHR	2690	0.4867	9.5129	3.815903e-02
ENSG00000174899	C3orf55	152078	0.4883	5.4867	3.111005e-02
ENSG00000101751	POLI	11201	0.4884	7.0996	2.656051e-02
ENSG00000131844	MCCC2	64087	0.4884	6.0985	3.200586e-02
ENSG00000083750	RRAGB	10325	0.4898	7.2221	2.340420e-02
ENSG00000077063	CTTNBP2	83992	0.4909	4.5568	3.706394e-02
ENSG00000011007	TCEB3	6924	0.4939	5.8175	3.693537e-02
ENSG00000115361	ACADL	33	0.4948	6.1097	4.324927e-03
ENSG00000173611	SCAI	286205	0.4959	4.6454	4.350849e-02
ENSG00000111142	METAP2	10988	0.4966	8.9505	4.526152e-02
ENSG00000176894	PGAM5	192111	0.4985	5.6986	1.502205e-02
ENSG00000176894	PXMP2	5827	0.4985	5.6986	1.502205e-02
ENSG00000080200	CRYBG3	131544	0.5017	9.3391	3.330015e-02
ENSG00000139631	CSAD	51380	0.5065	7.1409	2.767380e-02
ENSG00000122912	SLC25A16	8034	0.5096	8.5186	1.946357e-02
ENSG00000112715	VEGFA	7422	0.5114	5.1310	7.581990e-03
ENSG00000198894	KIAA1737	85457	0.5120	6.8701	9.034694e-03
ENSG00000062725	APPBP2	10513	0.5132	7.2189	9.557316e-03
ENSG00000175198	PCCA	5095	0.5145	8.5949	5.122069e-03
ENSG00000171503	ETFDH	2110	0.5156	7.5380	1.635653e-02
ENSG00000143994	ABHD1	84696	0.5181	4.4764	2.945335e-02
ENSG00000197885	NKIRAS1	28512	0.5223	5.8450	4.126641e-02
ENSG00000136521	NDUFB5	4711	0.5231	9.7093	3.786032e-02
ENSG00000166200	COPS2	9318	0.5291	7.0577	8.341510e-03
ENSG00000102471	NDFIP2	54602	0.5291	5.9254	1.680781e-02
ENSG00000121310	ECHDC2	55268	0.5299	6.6478	1.856304e-02
ENSG00000175879	HOXD8	3234	0.5300	6.9886	3.269018e-02
ENSG00000181092	ADIPOQ	9370	0.5314	10.9475	1.353540e-02
ENSG00000114054	PCCB	5096	0.5322	7.1138	4.694557e-02
ENSG00000100714	MTHFD1	4522	0.5324	9.6294	4.082911e-03

ENSG00000102760	C13orf15	28984	0.5354	10.8688	1.954289e-02
ENSG00000169714	CNBP	7555	0.5372	9.1731	2.107862e-02
ENSG00000169946	ZFPM2	23414	0.5373	6.9204	2.605359e-02
ENSG00000164167	LSM6	11157	0.5383	9.4333	6.644722e-03
ENSG00000083720	OXCT1	5019	0.5405	7.3908	4.098102e-02
ENSG00000113580	NR3C1	2908	0.5428	9.1156	1.720590e-02
ENSG00000166012	TAF1D	79101	0.5447	6.2606	6.525967e-04
ENSG00000173917	HOXB2	3212	0.5455	7.3395	3.915133e-02
ENSG00000198270	TMEM116	89894	0.5458	5.6950	2.721002e-02
ENSG00000104765	BNIP3L	665	0.5572	10.4982	2.064702e-03
ENSG00000083123	BCKDHB	594	0.5575	7.0517	6.104579e-03
ENSG00000166228	PCBD1	5092	0.5618	8.0450	8.294676e-03
ENSG00000060762	BRP44L	51660	0.5622	8.0129	2.702214e-02
ENSG00000152795	HNRPDL	9987	0.5631	7.7712	2.546932e-02
ENSG00000154719	MRPL39	54148	0.5673	7.0570	7.069165e-03
ENSG00000119801	YPEL5	51646	0.5695	9.8201	3.442499e-03
ENSG00000182919	C11orf54	28970	0.5698	6.3916	1.000574e-02
ENSG00000181784	RNASE4	6038	0.5762	9.8517	4.356177e-03
ENSG00000125375	ATP5S	27109	0.5763	6.9771	1.308470e-03
ENSG00000112245	PTP4A1	7803	0.5787	6.7398	1.705363e-02
ENSG00000127955	GNAI1	2770	0.5799	8.5668	9.853692e-03
ENSG00000109738	GLRB	2743	0.5823	4.6905	1.910496e-02
ENSG00000133317	LGALS12	85329	0.5824	10.1091	3.027844e-02
ENSG00000180530	NRIP1	8204	0.5863	8.5466	2.532381e-02
ENSG00000100380	FAM10A4	145165	0.5864	9.5975	1.523973e-02
ENSG00000100380	ST13	6767	0.5864	9.5975	1.523973e-02
ENSG00000154640	BTG3	10950	0.5872	8.4807	2.838315e-02
ENSG00000110871	COQ5	84274	0.5881	7.0176	1.176001e-02
ENSG00000132541	HRSP12	10247	0.5949	8.9770	8.303163e-03
ENSG00000169738	DCXR	51181	0.6001	8.4515	8.149197e-03
ENSG00000189184	PCDH18	54510	0.6046	6.6843	2.383571e-02
ENSG00000130414	NDUFA10	4705	0.6053	8.0632	1.255367e-04
ENSG00000171862	PTEN	5728	0.6080	9.0651	1.034524e-02
ENSG00000123472	ATPAF1	64756	0.6089	8.8516	3.680905e-04
ENSG00000185808	PIGP	51227	0.6100	8.7743	1.544391e-02
ENSG00000049759	NEDD4L	23327	0.6116	5.9765	3.664534e-02
ENSG00000100711	ZFYVE21	79038	0.6223	6.6034	1.119205e-02
ENSG00000108278	ZNHIT3	9326	0.6235	7.4952	8.148200e-03
ENSG00000148090	AUH	549	0.6252	6.5929	1.137927e-02
ENSG00000140740	UQCRC2	7385	0.6264	9.8653	8.863638e-03
ENSG00000113971	NPHP3	27031	0.6266	6.0645	2.906068e-02
ENSG00000181938	GINS3	64785	0.6298	5.6996	1.378339e-02
ENSG00000182118	FAM89A	375061	0.6301	8.5928	1.635653e-02
ENSG00000111276	CDKN1B	1027	0.6305	7.3990	1.219098e-02

ENSG00000132561	MATN2	4147	0.6311	8.6675	1.836823e-02
ENSG00000166347	CYB5A	1528	0.6315	11.2667	1.408618e-02
ENSG00000138796	HADH	3033	0.6323	10.9082	5.507830e-04
ENSG00000133028	SCO1	6341	0.6325	7.1954	1.441791e-02
ENSG00000165269	AQP7	364	0.6353	7.1929	1.236089e-02
ENSG00000196616	ADH1B	125	0.6395	11.9002	1.552756e-02
ENSG00000123080	CDKN2C	1031	0.6421	10.3984	8.897199e-03
ENSG00000050426	LETMD1	25875	0.6453	6.3727	8.223722e-03
ENSG00000156171	DRAM2	128338	0.6456	9.4542	2.052925e-02
ENSG00000079974	RABL2B	11158	0.6622	7.7575	1.494214e-02
ENSG00000185418	TARSL2	123283	0.6677	6.2674	2.810510e-02
ENSG00000173599	PC	5091	0.6678	5.1467	3.864050e-02
ENSG00000008311	AASS	10157	0.6744	4.5777	2.745314e-02
ENSG00000107372	ZFAND5	7763	0.6772	8.2529	3.776940e-02
ENSG00000164331	ANKRA2	57763	0.6829	4.9353	1.306437e-02
ENSG00000107186	MPDZ	8777	0.6853	7.9352	1.635653e-02
ENSG00000117791	MOSC2	54996	0.6919	8.4993	1.110010e-03
ENSG00000106049	HIBADH	11112	0.6928	8.7354	3.735242e-03
ENSG00000116761	CTH	1491	0.6976	3.9061	2.188747e-02
ENSG00000147592	LACTB2	51110	0.7088	7.8561	1.635653e-02
ENSG00000153291	SLC25A27	9481	0.7168	5.1049	4.153419e-02
ENSG00000197442	MAP3K5	4217	0.7220	7.7200	4.432520e-02
ENSG00000136383	ALPK3	57538	0.7285	4.9521	1.991699e-02
ENSG00000135821	GLUL	2752	0.7303	7.0100	7.051981e-04
ENSG00000138640	FAM13A	10144	0.7324	6.7454	1.669762e-02
ENSG00000204789	ZNF204P	7754	0.7326	6.1594	4.121332e-03
ENSG00000159596	TMEM69	51249	0.7370	6.3528	2.884646e-02
ENSG00000177485	ZBTB33	10009	0.7391	6.2328	2.126521e-02
ENSG00000123612	ACVR1C	130399	0.7400	7.8731	2.767834e-03
ENSG00000140374	ETFA	2108	0.7446	9.3845	5.122069e-03
ENSG00000204228	HSD17B8	7923	0.7472	6.8926	2.127045e-03
ENSG00000100417	PMM1	5372	0.7492	7.5848	4.109627e-02
ENSG00000205581	HMGN1	3150	0.7501	8.5399	1.955438e-02
ENSG00000170525	PFKFB3	5209	0.7520	5.8441	1.454460e-02
ENSG00000076555	ACACB	32	0.7528	8.2977	1.717074e-03
ENSG00000134463	ECHDC3	79746	0.7564	10.5544	1.335997e-03
ENSG00000144749	LRIG1	26018	0.7764	6.2608	1.353650e-03
ENSG00000144891	AGTR1	185	0.7849	7.1353	2.931448e-02
ENSG00000152642	GPD1L	23171	0.7871	5.7092	7.552883e-03
ENSG00000100077	ADRBK2	157	0.7909	7.8003	2.560135e-02
ENSG00000087338	GMCL1	64395	0.7967	5.5078	2.702214e-02
ENSG00000087338	GMCL1L	64396	0.7967	5.5078	2.702214e-02
ENSG00000109339	MAPK10	5602	0.7988	7.1950	2.634255e-03
ENSG00000005249	PRKAR2B	5577	0.7988	8.8142	3.956396e-02

ENSG00000144161	ZC3H8	84524	0.7995	4.6991	3.387835e-02
ENSG00000111231	GPN3	51184	0.8006	8.0754	1.454460e-02
ENSG00000182902	SLC25A18	83733	0.8172	6.2066	1.388978e-02
ENSG00000135917	SLC19A3	80704	0.8192	9.4789	1.955438e-02
ENSG00000160972	PPP1R16A	84988	0.8285	6.6369	1.406219e-02
ENSG00000103528	SYT17	51760	0.8302	4.6373	3.553720e-02
ENSG00000131831	RAI2	10742	0.8355	6.5890	9.444753e-03
ENSG00000179941	BBS10	79738	0.8470	5.6393	2.192821e-03
ENSG00000180660	MAB21L1	4081	0.8497	6.5648	2.049037e-02
ENSG00000064989	CALCRL	10203	0.8517	6.8066	6.021482e-03
ENSG00000172057	ORMDL3	94103	0.8608	7.1373	2.064702e-03
ENSG00000035664	DAPK2	23604	0.8792	7.1080	1.556103e-02
ENSG00000140876	NUDT7	283927	0.8893	6.9980	1.635653e-02
ENSG00000122691	TWIST1	7291	0.9010	8.9625	4.103600e-03
ENSG00000134324	LPIN1	23175	0.9030	7.8906	2.130406e-02
ENSG00000138835	RGS3	5998	0.9067	6.1180	1.819997e-02
ENSG00000171723	GPHN	10243	0.9073	6.7898	2.906068e-02
ENSG00000077616	NAALAD2	10003	0.9102	4.2832	3.622800e-02
ENSG00000170293	CMTM8	152189	0.9106	5.8590	2.297142e-02
ENSG00000115425	PECR	55825	0.9175	8.0759	6.865986e-04
ENSG00000183807	FAM162B	221303	1.0124	6.9387	2.276328e-02
ENSG00000137941	TTLL7	79739	1.0137	4.7194	3.191783e-02
ENSG00000111058	ACSS3	79611	1.0334	7.4732	1.578617e-03
ENSG00000176194	CIDEA	1149	1.0456	9.9387	5.742405e-03
ENSG00000011198	ABHD5	51099	1.0742	7.9895	1.557149e-02
ENSG00000164708	PGAM2	5224	1.1092	5.0332	3.104759e-02
ENSG00000105516	DBP	1628	1.1554	5.4195	4.811892e-02
ENSG00000109929	SC5DL	6309	1.1600	7.5952	2.694681e-02
ENSG00000073605	GSDMB	55876	1.1723	7.3634	2.351075e-02
ENSG00000163536	SERPINI1	5274	1.1932	7.2777	1.441148e-02
ENSG00000118729	CASQ2	845	1.2182	5.2836	3.648389e-02
ENSG00000173253	DMRT2	10655	1.2880	6.0896	1.556103e-02
ENSG00000165028	NIPSNAP3B	55335	1.3566	8.1276	1.036294e-02
ENSG00000185100	ADSSL1	122622	1.4063	7.0922	3.960058e-02
ENSG00000160862	AZGP1	563	1.4214	8.5647	2.632655e-02
ENSG00000165730	STOX1	219736	1.4906	5.7303	4.906514e-03
ENSG00000206384	COL6A6	131873	1.6500	5.7137	2.453060e-02
ENSG00000084674	APOB	338	1.7421	6.6162	2.160066e-03
ENSG00000134548	C12orf39	80763	2.4570	6.9310	2.906068e-02
ENSG00000164879	CA3	761	2.7838	6.0856	1.666361e-02

Figure S1. Protein expression in the skeletal muscle related to mitochondrial energy metabolism, glucose uptake and insulin signaling. Values are reported as mean and the error bars represent standard deviation.

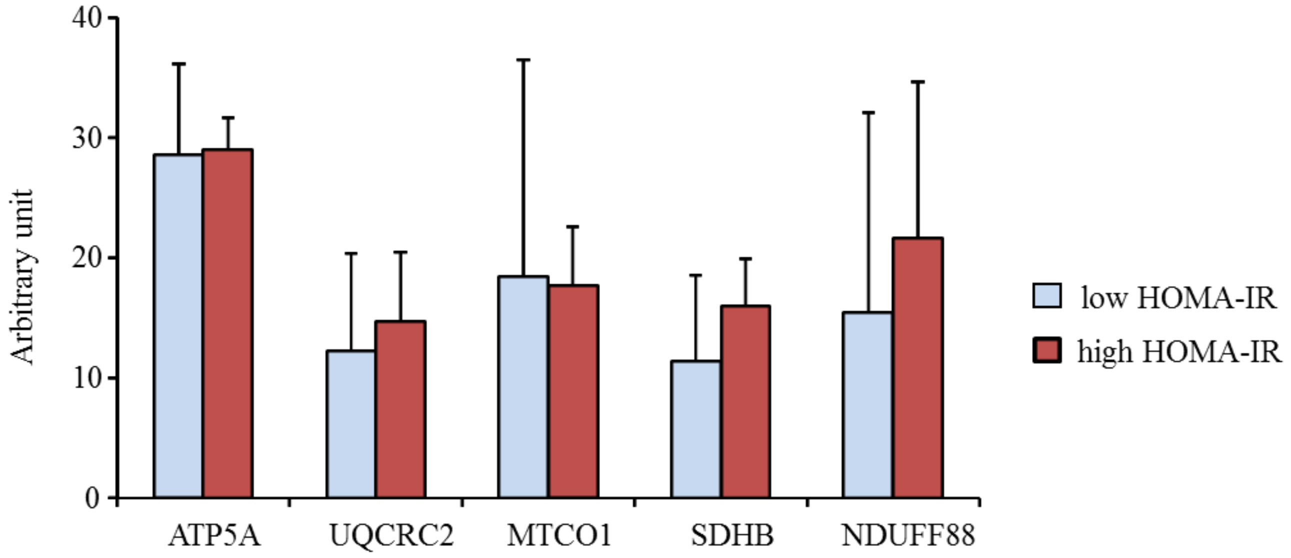

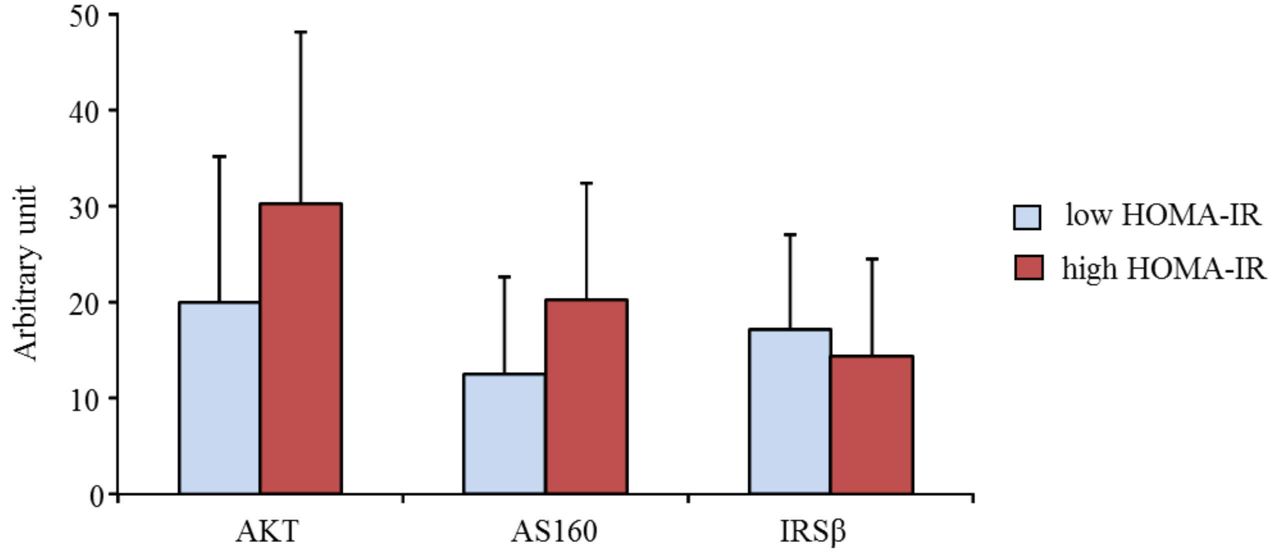

Supplement: Supplementary Information [file srep24540-s1.pdf]
